# Resveratrol delays the progression of diabetic nephropathy through multiple pathways: A dose–response meta‐analysis based on animal models

**DOI:** 10.1111/1753-0407.13608

**Published:** 2024-09-12

**Authors:** Xiaojing Liu, Xia Gu, Jiao Zhang, Xiangmeng Li, Xiansen Wei, Shimin Jiang, Wenge Li

**Affiliations:** ^1^ Graduate School of Peking Union Medical College, Chinese Academy of Medical Sciences & Peking Union Medical College Beijing China; ^2^ Department of Nephrology China‐Japan Friendship Hospital (Institute of Clinical Medical Sciences) Beijing China; ^3^ Beijing University of Chinese Medicine Beijing China

**Keywords:** animal model, diabetic nephropathy, meta‐analysis, renal pathology, resveratrol, systematic review

## Abstract

**Objective:**

Accumulating experimental evidence has shown that resveratrol supplementation is effective for treating diabetic nephropathy (DN) in animal models. In this systematic review and meta‐analysis, we assessed the effects and multiple mechanisms of resveratrol in animal models of DN.

**Methods:**

Before September 2023, preclinical literature was systematically searched and screened across PubMed, Web of Science, EMBASE, and the Cochrane Library. Forty‐two studies were included, and the risk of bias tool from SYRCLE was used to assess the methodological quality. Pooled overall effect sizes of the results were generated by STATA 16.0.

**Results:**

The overall results provide preliminary evidence that the consumption of resveratrol can significantly reduce the mesangial index, glomerular basement membrane thickness, glomerular hypertrophy, serum creatinine, blood urea nitrogen, 24‐h urinary protein, blood glucose, kidney index, total cholesterol, triglyceride, and low‐density lipoprotein cholesterol levels. In contrast, the levels of albumin and high‐density lipoprotein cholesterol are significantly increased. However, resveratrol did not significantly reduce creatinine clearance or glycated hemoglobin levels. Dose–response analysis revealed that resveratrol was most effective at improving kidney function and reducing DN when administered at lower doses of ≤15 mg/kg/day or higher doses of 100–200 mg/kg/day, with significant improvements in biochemical kidney injury markers and a better effect on dysglycemia.

**Conclusions:**

The benefits of resveratrol in DN are likely due to its anti‐inflammatory, antioxidant, metabolic regulatory, and autophagy‐promoting effects. To confirm these findings for clinical use, further large‐scale, long‐term, high‐quality preclinical trials are warranted to accurately assess the anti‐DN effects and safety of resveratrol.

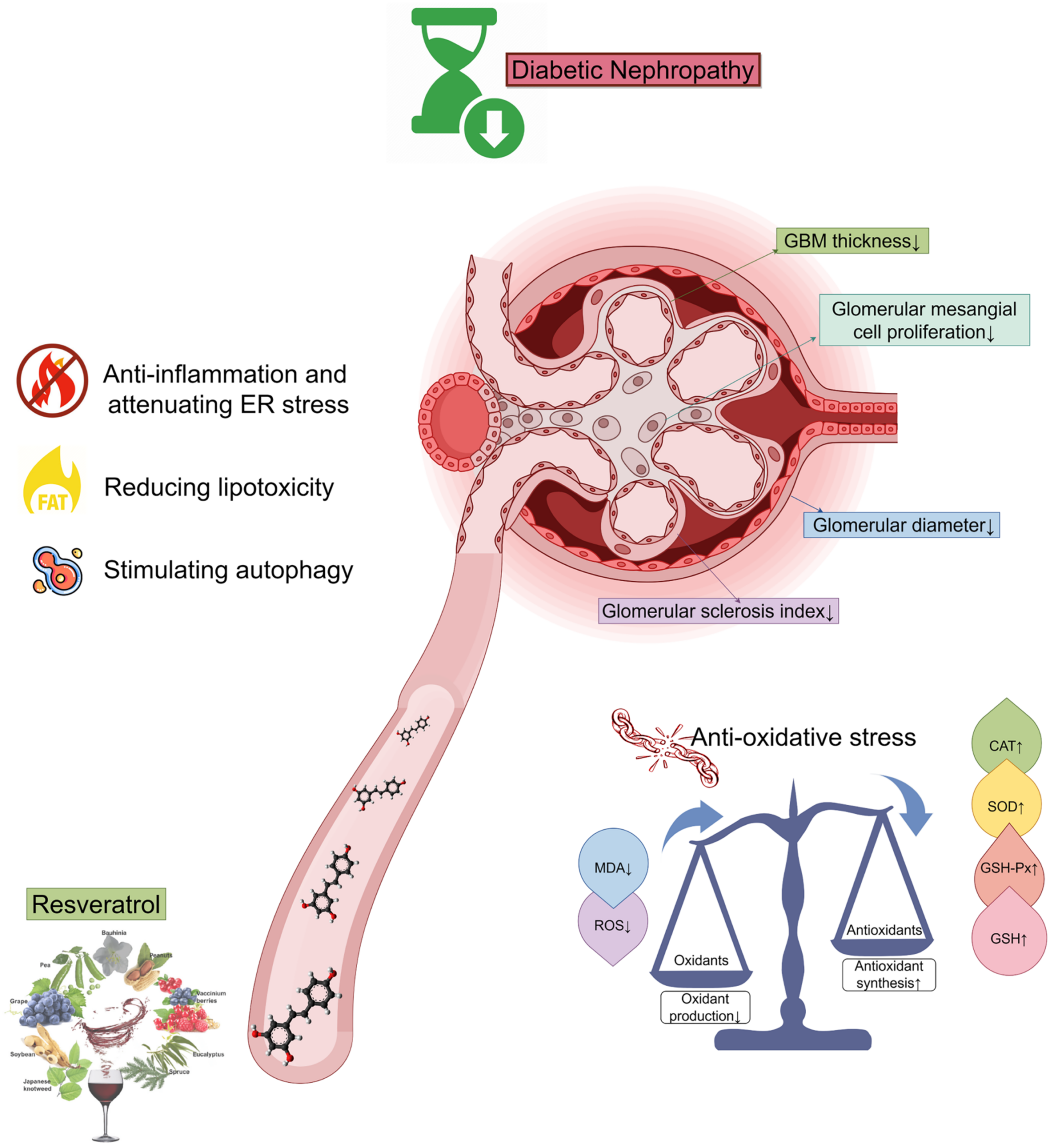

## Graphical abstract

- Resveratrol improves renal function in diabetic nephropathy at low and high doses.
- It targets oxidative stress, inflammation, and metabolic regulation for renoprotection.
- Further research is needed for clinical translation and optimal dosing strategies.

## 1 INTRODUCTION

Diabetic nephropathy (DN) is a significant health issue affecting nearly half of diabetic patients and often leads to end‐stage kidney disease, which requires treatments such as dialysis or transplantation. DN also significantly increases the risk of cardiovascular complications and mortality, underscoring its importance as a target for medical intervention and research.^1^

DN is a multifactorial disease primarily driven by hyperglycemia, inflammation, and oxidative stress.^2^ These factors, especially in streptozotocin (STZ)‐induced diabetic animal models, directly contribute to the deterioration of renal function and the onset of DN.^3^, ^4^ Despite these challenges, DN treatment has advanced. The standard treatment for DN includes renin‐angiotensin system inhibitors such as angiotensin‐converting enzyme inhibitors and angiotensin receptor blockers, which are effective for treating type 2 diabetes‐related DN but may increase the risk of acute kidney injury and hyperkalemia.^2^, ^5^ Emerging antidiabetic medications, such as sodium‐glucose cotransporter 2 (SGLT2) inhibitors, glucagon‐like peptide 1 receptor agonists, dipeptidyl peptidase‐4 (DPP‐4) inhibitors, mineralocorticoid receptor antagonists, and selective endothelin receptor antagonists, show promise but also have potential side effects.^6^, ^7^, ^8^, ^9^ Notably, SGLT2 inhibitors may increase the risk of diabetic ketoacidosis in patients with type 2 diabetes and kidney disease, whereas DPP‐4 inhibitors may increase the risk of venous thromboembolism. Given these challenges, exploring new therapeutic strategies is essential.^6^, ^9^

**FIGURE 1:**
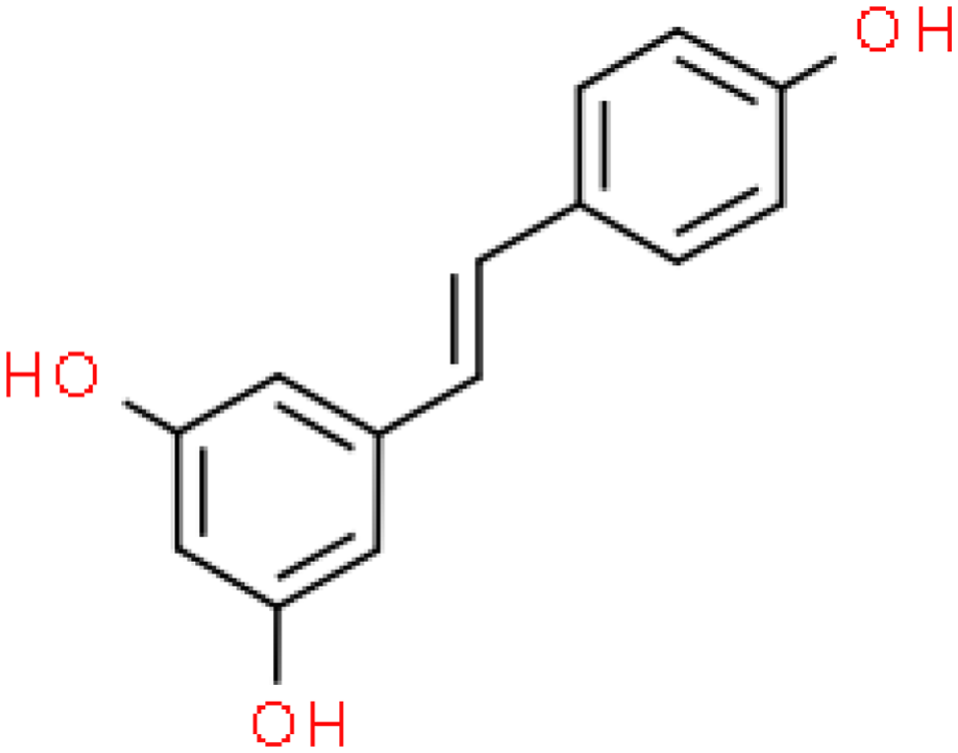
The chemical structure of resveratrol.

Emerging research indicates that natural compounds, particularly dietary polyphenols, may help prevent the progression of renal failure in DN patients.^10^, ^11^, ^12^, ^13^, ^14^ Resveratrol (C14H12O3, Figure 1), a natural polyphenolic phytochemical with health‐promoting bioactivities, is not only readily absorbed from dietary sources such as peanuts, peanut butter, grapes, and red wine but also a powerful constituent of *Polygonum cuspidatum*, or Japanese knotweed (Hu Zhang), further recognized for its multitude of health benefits.^15^ The polyphenolic structure of resveratrol, which confers anti‐inflammatory and antioxidant activity, has demonstrated significant health benefits in animal studies and may reduce oxidant‐induced apoptosis and low‐density lipoprotein (LDL) oxidation.^16^, ^17^, ^18^ These properties are crucial for addressing the inflammation and oxidative stress that contribute to the progression of DN. However, in the literature related to resveratrol treatment for DN, different investigators have focused on different indicators and reported differences in the efficacy of the same indicators. The scattered evidence, uncertainty of mechanisms, and adverse drug reactions add uncertainty and conflict to the hypothesis that resveratrol can ameliorate renal injury in diabetic animal models.

Preclinical research offers a deeper understanding of drug efficacy, enhancing the validity of experimental hypotheses. Systematic reviews and meta‐analyses are essential tools for refining multimodal treatment strategies. We performed a systematic review and meta‐analysis of rodent studies on resveratrol therapy for DN following the Preferred Reporting Items for Systematic Reviews and Meta‐Analysis (PRISMA) criteria to comprehensively assess its effects. The evidence from studies on resveratrol is crucial for both healthy individuals and those with DN. It underscores the potential of resveratrol for DN prevention and treatment, with implications for personalized medicine. The research also informs the development of optimal dosing strategies and enhances our understanding of resveratrol's multifaceted mechanisms, which can guide both lifestyle choices and clinical interventions.

The key clinical questions we reviewed and addressed in our study included the following: (1) evaluation of the efficacy of resveratrol on morphometric parameters, kidney functional parameters, metabolic parameters, and biochemical parameters in animal models of DN; (2) assessment of the safety of resveratrol in DN; (3) assessing the benefits of different doses; (4) review of the mechanisms of resveratrol in treating DN; (5) summarizing of the quality and limitations of existing animal studies; and (6) evaluation of the current evidence regarding whether resveratrol has clinical translational value for the treatment of DN.

## 2 METHODS

This systematic review and meta‐analysis were performed according to the Cochrane Handbook for Systematic Reviews of Interventions and reported based on the PRISMA guidelines. The protocol for this systematic review and meta‐analysis is available on the PROSPERO website (CRD42024434579).

### 2.1 Search strategy and study selection

Electronic bibliographic databases such as PubMed, EMBASE, Web of Science, and the Cochrane Library were searched for relevant rodent studies published from January 2010 to September 2023 to assess the effects of resveratrol on kidney outcomes or other default outcomes. Manual searches within the references of the included studies were carried out to identify other studies that could potentially be included. The language was limited to English. Literature search strategies were developed using terms related to resveratrol and DN. Resveratrol‐related terms included “resveratrol,” “3,5,4′‐trihydroxystilbene,” “4′,5‐trihydroxystilbene,” “3,4′,5‐stilbenetriol,” “trans‐resveratrol‐3‐O‐sulfate,” “trans‐resveratrol 3° sulfate,” “SRT 501,” “SRT501,” “SRT‐501,” “501–36‐0,” “cis‐resveratrol,” “cis Resveratrol,” “trans‐resveratrol,” “trans Resveratrol,” “Resveratrol‐3‐sulfate,” and “Resveratrol 3 sulfate.” The DN‐related terms included “diabetic nephropathy” or “diabetic nephropathy,” “diabetic renal disease,” “kidney diseases, diabetic,” “kidney disease, diabetic,” “diabetic kidney diseases,” “diabetic kidney disease,” “nephropathies, diabetic,” “diabetic nephropathy,” “nephropathy, diabetic,” “DKD,” and “DN.” In addition, the reference lists of the included publications were also manually scrutinized to obtain additional trials. The specific search strategies used for each database are summarized in Supporting Information Appendix S1. For abbreviations, see Supporting Information Appendix S2.

### 2.2 Eligibility criteria

The criteria for selecting articles were as follows: (1) population (P): rodent models of DN were established in a generally accepted manner; (2) intervention (I) and control (C): the experimental group received monotherapy with resveratrol at any dose, and the model group received equivalent amounts of nonfunctional substances or no treatment; and (3) outcome (O). Primary outcomes included alterations in histopathology, morphometry, and renal function, specifically serum creatinine (Scr), blood urea nitrogen (BUN), and creatinine clearance (CCr), along with 24‐h urinary protein levels (24hUTP). Secondary outcomes assessed the renoprotective effects of resveratrol on DN, focusing on metabolic and biochemical changes, such as blood glucose (BG) and hemoglobin A1c (HbA1c), body weight (BW), kidney weight (KW), kidney index (KI), systolic blood pressure (SBP), total cholesterol (TC), triglycerides (TG), LDL cholesterol (LDL‐C), and high‐density lipoprotein cholesterol (HDL‐C).

The exclusion criteria were as follows: (1) not an original full research paper (e.g., review, editorials/letters, abstracts); (2) animals with comorbidities, ex vivo, in vitro, or in silico models; (3) intervention: resveratrol without batch number; (4) control: other preparations of resveratrol (some pharmaceutical preparation or nutritional products containing resveratrol); (5) combination therapy; (6) study design: case studies, crossover studies, and studies without a separate control group; (7) no relevant outcome measures reported; (8) duplicate publication; and (9) studies without full text.

### 2.3 Data extraction

Two reviewers independently evaluated all the eligible articles fulfilling the following data extraction criteria: (1) study ID: the first author's name and the publication year; (2) characteristics and data regarding rodents: age, total number (*N*), size in each group, species (mice/rats), strains, sex (male/female), and weight; (3) model‐establishing details: the methods and criteria for disease modeling successfully; (4) intervention and control features: the regimen (dosage levels, administration frequency and period, drug delivery route, name, and volume of vehicle) in therapeutic and model groups; (5) outcomes and partial corresponding *p* values; and (6) related mechanisms underlying the action of resveratrol against DN in each study. The list of the variables for which data were sought is found in Table 1.

**TABLE 1:** Variables for which data were sought.

P
Age
Total number (*N*)
Size in each group
Species (mice/rats)
Strains
Sex (male/female)
Weight
The methods and criteria for disease modeling successfully

I
Single/total dosage levels
Administration frequency
Administration period

C
Name of vehicle

O
Thickness of the glomerular basement membrane (GBM)
The glomerular sclerosis index (GSI)
Glomerular mesangial cell proliferation
Serum creatinine (Scr)
Blood urea nitrogen (BUN)
Creatinine clearance (CCr)
24‐h urinary protein levels (24hUTP)
24‐h urinary albumin excretion rate (UAER)
The urine albumin‐to‐creatinine ratio (UACR)
24‐h urinary microalbumin (UMA)
Urine volume
Alb
Blood glucose (BG)
Hemoglobin A1c (HbA1c)
Body weight (BW)
Kidney weight (KW)
Kidney index (KI)
Systolic blood pressure (SBP)
Total cholesterol (TC)
Triglycerides (TG)
Low‐density lipoprotein cholesterol (LDL‐C)
*p* values
High‐density lipoprotein cholesterol (HDL‐C)

*Note*: *p* values and data were extracted from the highest drug doses and used data from the final time point for each group when multiple were reported.

For a comprehensive analysis across varying doses and outcomes, we focused on extracting the *p* values and data specifically from the highest drug doses. In instances where multiple time points were reported for each group's outcomes, we selected the data from the final time point, corresponding to the longest duration of observation, for our analysis. For all study outcomes, quantitative details were extracted when data were available in at least two different articles. Regarding continuous variables of interest, the mean score and standard deviation from both the treatment and model groups were measured from related graphs with the publicly available WebPlotDigitizer, where the outcomes were only rendered graphically but not as numerical text.

### 2.4 Risk of bias and quality of evidence

The methodological quality of the included studies was assessed according to the Systematic Review Center for Laboratory Animal Experimentation Risk of Bias (SYRCLE's RoB) tool. The SYRCLE RoB tool for animal experiments contains 10 entries based on six types of bias: (1) sequence generation, (2) baseline characteristics, (3) allocation concealment, (4) random housing, (5) blinding, (6) random outcome assessment, (7) blinding, (8) incomplete outcome data, (9) selective outcome reporting, and (10) other sources of bias. The results of the assessments are “yes,” “no,” and “unclear,” representing “low risk of bias,” “high risk of bias,” and “insufficient details have been reported to assess the risk of bias properly”.^19^ The GRADE evidence quality assessment tool was applied to assess confidence. Each qualitative outcome was rated as high, medium, low, or very low quality of evidence, according to five dimensions lowering quality (risk of bias, inconsistency, indirectness, imprecision, and publication bias) and three dimensions enhancing quality (large magnitude of effect, dose–response, and confounders likely minimize the effect).

Two reviewers independently performed the quality assessment, and discrepancies were discussed with a third reviewer.

### 2.5 Quantitative synthesis and statistical analyses

The STATA version 16.0 software was utilized for all meta‐analyses where possible, and a *p* value <0.05 (*p* < 0.05) indicated statistical significance. The summary statistics of the outcomes were quantitatively determined using the standardized mean difference (SMD) with the corresponding 95% confidence interval (95% CI). We tested interstudy heterogeneity and the degree of inconsistency using both the *I*‐square (*I* ^2^) and *χ* ^2^ tests as the evaluation indices (random effects model [*I* ^2^ > 50%] or a fixed effects model [*I* ^2^ ≤ 50%]). When *I* ^2^ was above 50%, the result was considered to have a high level of heterogeneity. To explore potential causes of considerable inconsistency, we conducted a subgroup analysis to assess the source of heterogeneity in the included studies based on different rodent species (rats, mice), various methods of modeling (STZ/Alloxan, genetic mutation), varying single doses of resveratrol (low <20 mg/kg, 20 ≤ medium <100 mg/kg, high ≥100 mg/kg), varying single doses of resveratrol (low <100 mg/kg, 100 ≤ medium <500 mg/kg, high ≥500 mg/kg), and treatment duration (intervention start time) (<8 weeks, ≥ 8 weeks). Sensitivity analysis was conducted to evaluate whether a single study affects the overall effect sizes by removing one study at each stage. Publication bias was detected using Egger's test (|*t*| < 0.05 was considered to indicate publication bias). The relationship between the dose of resveratrol and the main DN index was modeled using radar maps.

## 3 RESULTS

### 3.1 Research screening

**FIGURE 2:**
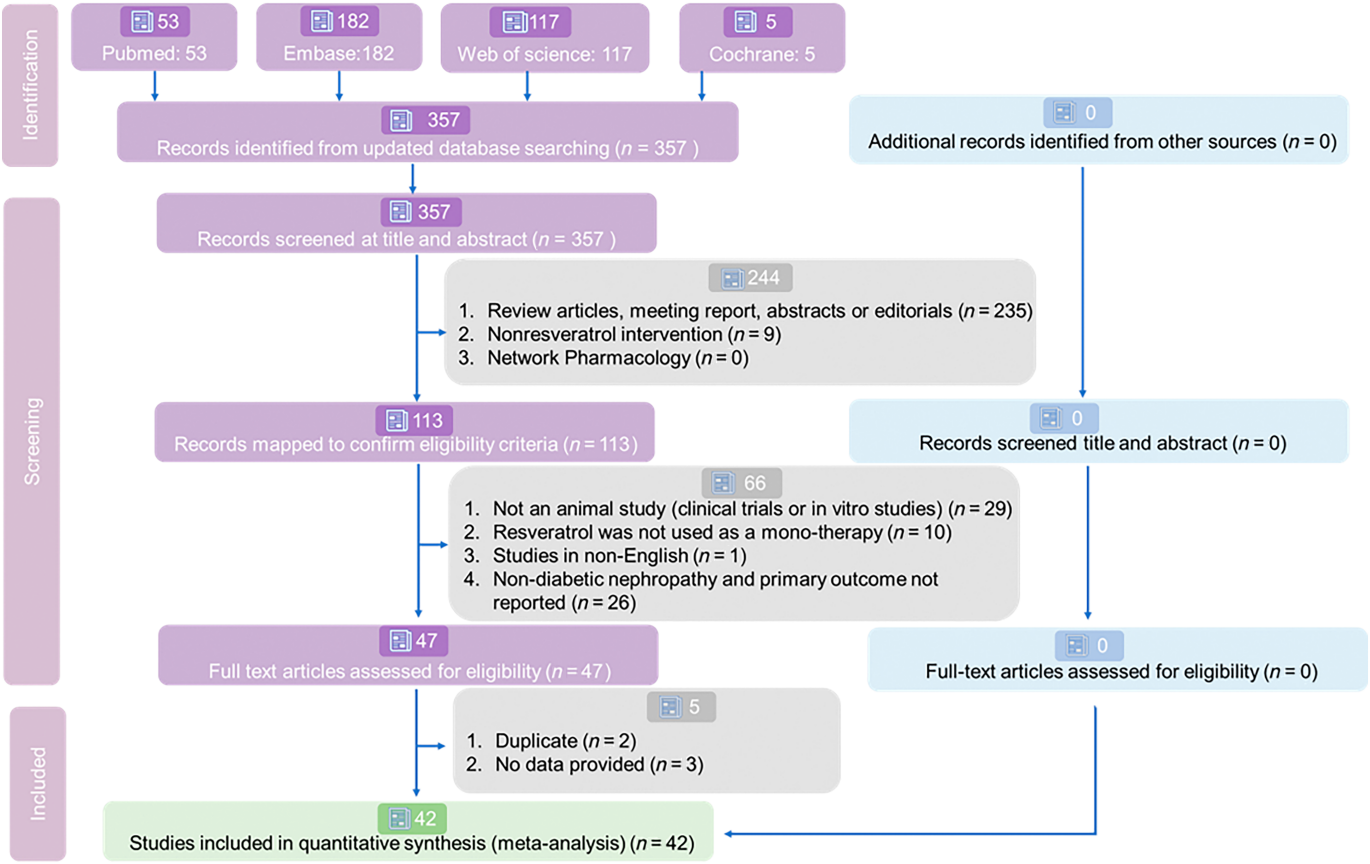
Chart of selection process following selection criteria.

After a comprehensive search, we identified 53 records from PubMed, 182 from EMBASE, 117 from Web of Science, and none from the Cochrane Library for systematic review and meta‐analysis. For the detailed selection process, see Figure 2. Ultimately, 42 eligible English studies met our inclusion criteria for this quantitative synthesis.^18^, ^20^, ^21^, ^22^, ^23^, ^24^, ^25^, ^26^, ^27^, ^28^, ^29^, ^30^, ^31^, ^32^, ^33^, ^34^, ^35^, ^36^, ^37^, ^38^, ^39^, ^40^, ^41^, ^42^, ^43^, ^44^, ^45^, ^46^, ^47^, ^48^, ^49^, ^50^, ^51^, ^52^, ^53^, ^54^, ^55^, ^56^, ^57^, ^58^, ^59^, ^60^ The review was reported according to PRISMA guidelines (Supporting Information Appendix S3: PRISMA checklist).

### 3.2 Characteristics of the included studies

**FIGURE 3:**
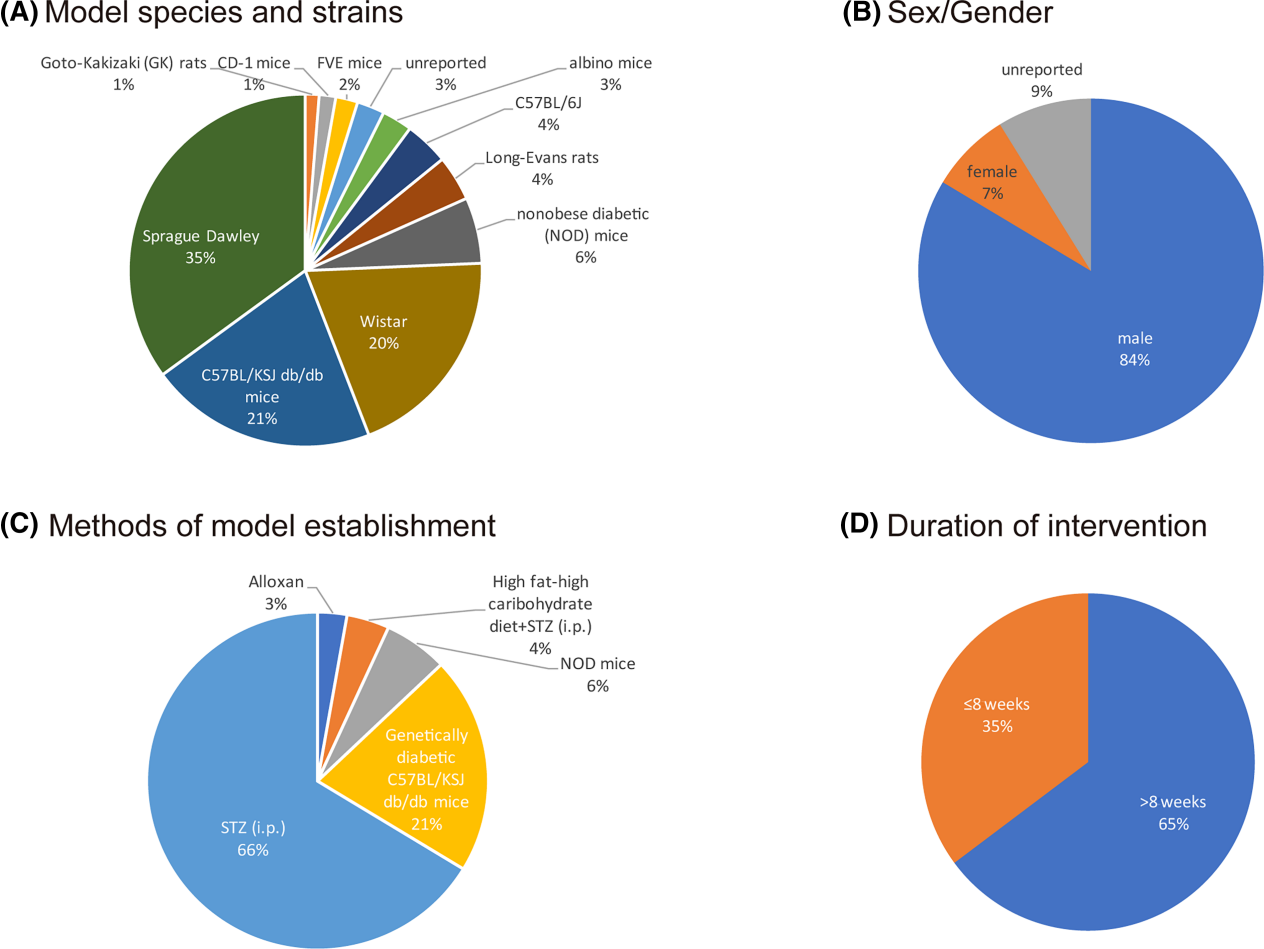
Study characteristics of eligible studies. Distribution of (A) model species and strains, (B) sex/gender, (C) methods of model establishment, and (D) duration of intervention.

The 42 articles contained a total of 793 animals from the treatment group exposed to resveratrol versus the model group that received a placebo or vehicle. The detailed characteristics of the included studies are shown in Table 2. The following strains were tested in rodent models with various species and strains (Figure 3A): 62.8% (478/793) in rats; 35.1% in Sprague–Dawley (278/793) rats; 19.8% in Wistar (157/793) rats; 4.2% in Long‐Evans (33/793) rats; 1.3% in Goto‐Kakizaki rats (10/793); 20.8% (165/793) in C57BL/KSJ db/db mice; 4.0% in C57BL/6J (32/793) mice; and 6.1% in nonobese diabetic (NOD) mice (48/793).

**TABLE 2:** Characteristics of the included studies.

Study, year	*N* = (T, C)	Species	Weight (g)	Age (weeks)	Animals model of DN	RSV dose mg/kg/day)	Duration weeks)	Control	Outcome index
Sharma et al. (2006)	7, 7	Rats	200–250	NR	STZ, 65 mg/kg	5, 10	2	NT	1. Scr 2. BUN 3. 24hUTP 4. urine volume 5.CCr 6. BG 7. BW 8. SBP
Ding et al. (2010)	6, 7	Rats	NR	10	STZ, 60 mg/kg	10	4	1% DMSO	1. Scr 2. 24hUTP 3. BG 4. BW 5. KW 6. KI 7. glomerular diameter
Chang et al. (2011)	26, 7	Rats	220–250	6–7	STZ, 65 mg/kg	1	1	Saline	1. Scr 2. BUN 3. BG 4. BW 5. TC 6. TG
Chen et al. (2011)	7, 7	Rats	NR	8–10	STZ, 65 mg/kg	0.75	8	Saline	1. Scr 2. 24hUTP 3. urine volume 4. BG 5. BW 6.KW 7.KI 8.TC 9.TG 10. glomerular diameter
Palsamy et al. (2011)	6, 6	Rats	160–180	6	STZ, 50 mg/kg	5	4	NT	1. CCr 2. BG 3.BW 4.KW
Khamneh et al. (2012)	6, 6	Rats	320–350	NR	STZ, 50 mg/kg	5	16	NT	1. Scr 2. BUN 3. BG 4. HbAlc 5. BW
Wu et al. (2012)	12, 10	Rats	200–240	NR	STZ, 60 mg/kg	30	12	Saline	1. Scr 2.BUN 3. 24hUTP 4. BG 5.KI 6.BW
Ramar et al. (2012)	6, 6	Mice	20–25	NR	Alloxan, 75 mg/kg	20	2	NT	1. Scr 2. BUN 3. BG
Kim et al. (2013)	8, 8	Mice	NR	6	db/db mice	20	12	0.5% CMC	1.24hUTP 2. BG 3. HbAlc 4.CCr 5.BW 6.KW 7.SBP 7. mesangial area ratio
Huang et al. (2013)	8, 8	Rats	220	NR	STZ, 50 mg/kg	150	12	1% CMC	1. Scr 2.BUN 3. 24hUTP 4. BG 5.BW 6.KI
Jiang et al. (2013)	8, 8	Rats	200–220	10	STZ, 55 mg/kg	20	8	NT	1. Scr 2. BG 3.KI 4. mesangial area ratio
Wen et al. (2013)	25, 25	Rats	180–200 g	NR	STZ, 50 mg/kg	20	8	Saline.	1. 24hUTP 2.CCr 3. BG 4.BW 5.KW 6.GBM 7. glomerular diameter

Elbe et al. (2014)	7, 7	Rats	300–350	NR	STZ, 45 mg/kg	10	4	Saline	1. Scr 2. BUN 3. BG 4. BW 5. KW 6. GSI
Xu et al. (2014)	8, 8	Mice	26–30	8	STZ, 50 mg/kg	10	12	Saline	1.Scr 2.BUN 3. 24hUTP 4.BG 5.KI
Zhou et al. (2014)	10, 10	Rats	180–220 g	NR	STZ, 55 mg/kg	50	8	Saline	1.24hUTP 2. urine volume 3.CCR 4. glomerular diameter
Yaylali et al. (2015)	6, 6	Rats	200–250	NR	STZ, 50 mg/kg	10	2	NT	1. BG 2.BW
He et al. (2016)	8, 8	Mice	NR	9	db/db mice	40	12	NT	1. Scr 2. 24hUTP 3. BG 4.BW 5.KW 6. mesangial area ratio
Hussein et al. (2016)	9, 9	Rats	180–220	10	STZ, 60 mg/kg	5	8	NT	1. Scr 2. 24hUTP 3. CCr 4. BG 5. HbAlc 6.BW 7.KI
Wang, et al. (2016)	8, 8	Rats	250–280	8	STZ, 60 mg/kg	30	16	0.5% CMC	1. 24hUTP 2. CCr 3. BG 4.KI 5.GSI
Park et al. (2016)	8, 8	Mice	NR	6	db/db mice	20	12	0.5% CMC	1. Scr 2. 24hUTP 3. urine volume 4. BG 5.HbAlc 6.BW 7.KW mesangial area ratio
Yan et al. (2016)	8, 8	Mice	NR	12	db/db mice	40	12	0.5% CMC	1. Scr 2.BUN 3. 24hUTP 4. BG 5.BW 6.KW 7.KI
Huang et al. (2017)	6, 6	Mice	NR	8	db/db mice	10	12	Saline	1. BUN 2. 24hUTP 3. BG 4.BW 5.KW 6.KI 7.SBP
Qiao et al. (2017)	10, 10	Rats	230–270	8	STZ, 55 mg/kg	20	4	DMSO	1.24hUTP 2.urine volume 3.BG 4.BW 5.KW 6.KI
Xu et al. (2017)	8, 8	Mice	NR	8	db/db mice	100	12	0.5% CMC	1. Scr 2. 24hUTP 3. BG 4. glomerular diameter
Bashir et al. (2018)	10, 10	Rats	250–280	NR	STZ, 65 mg/kg	20 mg/kg	8	Saline	1. Scr 2.BUN 3.BG 4.HbAlc
Peng et al. (2018)	8, 8	Rats	NR	NR	STZ, 15 mg/kg	15	2	PBS	1. Scr 2. 24hUTP 3.BG 4.HbAlc 5.GSI
Maity et al. (2018)	6, 6	Rats	100–180	NR	STZ, 50 mg/kg	10	10	Saline	1. Scr 2. BUN 3.24hUTP 4. Alb 5. BG 6. BW 7. TC 8. HDL‐C
Yuan et al. (2018)	24, 24	Rats	200	NR	STZ, 50 mg/kg	50	6	0.5% CMC	1. Scr 2. BUN 3. 24hUTP 4. BG 5. KI 6.BW 7. TC 8. TG
Zhang et al. (2018)	6, 6	Mice	20–25	5	STZ, 140 mg/kg	30	4,8,12	0.5% CMC	1.BUN 2. 24hUTP 3. BG 4.TC
Rehman et al. (2018)	5, 5	Rats	180–250	NR	Alloxan, 120 mg/kg	30	4	NT	1. Scr 2.BUN 3.BG
Du et al. (2019)	6, 6	Mice	20–25	NR	High glucose‐fat diet+STZ (100 mg/kg)		8	0.5% CMC	1. Scr 2. BUN 3.BG 4. BW 5. KI 6.TG 7.LDL‐C 8. mesangial area ratio
Xian et al. (2019)	12, 15	Mice	20–24	8	NOD mice	200	8	NT	1. Scr 2.BUN 3. 24hUTP 4. BG 5.GBM
Xian et al. (2019)	11, 10	Mice	20–24	6–8	NOD mice		8	NT	1. Scr 2. BUN 3. 24hUTP 4. BG 5.BW 6.GBM 7.GSI
Cai et al. (2020)	7, 6	Mice	NR	8	db/db mice		12	Saline	1. BUN 2.24hUTP 3.BG 4.BW 5.KW 6.SBP
Du et al. (2020)	6, 6	Mice	30–35	7	db/db mice		2	0.5% CMC	1. Scr 2. BUN 3. BG 4. BW 5. KI
Szkudelska et al. (2020)	10, 10	Rats	NR	3	Goto‐Kakizaki (GK) rats	20	10	Saline	1. Scr 2. BUN 3. BG 4. Alb 5. TC 6. LDL‐C 7.HDL‐C
Wang et al. (2020)	13, 15	Mice	17–23	8	db/db mice	10	12	Saline	1. BUN 2. 24hUTP 3. BG 4.BW 5.KW 5. TC 6. glomerular diameter
Zhu et al. (2020)	10, 10	Rats	NR	6	STZ, 55 mg/kg	5, 20	8	Saline	1. Scr 2. BUN 3. 24hUTP 4. KI 5. TG 6. TC 7. LDL‐C 8. HDL‐C
Zhang et al. (2020)	10, 10	Mice	30–33	6	db/db mice	40	8,20	0.5%CMC	1. 24hUTP 2. BG 3.BW
Gu et al. (2021)	10, 10	Mice	22–26	6	High‐fat diet (HFD)	100	6	0.1% DMSO	1. Scr 2. BUN 3. BG 4. BW 5. TC 6.TG 7. LDL‐C 8.HDL‐C
Qi et al. (2021)	20, 20	Mice	NR	NR	STZ, 35 mg/kg	150	4	Saline	1.24hUTP 2. BG 3. TC 4. TG
Zhang et al. (2022)	10, 10	Rats	165–195	NR	STZ, 60 mg/kg	20	8	Water	1.24hUTP

Abbreviations: Scr, Serum creatinine; BUN, blood urea nitrogen; BG, blood glucose; CMC, carboxymethylcellulose; CCr, creatinine clearance rate; DMSO, dimethyl sulfoxide; 24hUTP, 24‐h urinary protein; BW, body weight; KW, kidney weight; KI, kidney index; GBM, glomerular basement membrane; GSI, glomerular sclerosis index; TC: Total Cholesterol; TG: Triglycerides; LDL‐C: low‐density lipoprotein cholesterol; HDL‐C: high‐density lipoprotein cholesterol; NOD: nonobese diabetic; NR, no report; NT, no treatment; STZ, streptozocin; PBS, phosphate buffer saline.

The sample size range was 10–50 laboratory individuals per study. Regarding sex (Figure 3B), male animals accounted for the predominant population (84%, 663/793), female animals accounted for 8% (60/793), and the remaining 9% of individuals' sex (70/793) was not specified. Four methods have been used to establish a DN model (Figure 3C), including intraperitoneal (i.p.) injection of STZ (66.0%, 525/793), a high‐fat/high‐carbohydrate diet+STZ (i.p.) (4.0%, 32/793), genetically diabetic C57BL/KSJ db/db mice (21.0%, 165/793), or NOD mice (6.1%, 48/793). The daily dose range of resveratrol was between 1 and 200 mg•kg^−1^, and resveratrol was administered via oral gavage, in an intragastric manner or via intraperitoneal (i.p.) injection. There were three dosage gradients (low <20 mg/kg, 20 ≤medium <100 mg/kg, high ≥100 mg/kg). Low doses were used in 15 studies (35.7%), medium doses were used in 23 studies (54.8%), and high doses were used in 4 studies (9.5%). Seven percent of the included studies (3/42) reported the use of multiple doses. The duration of the intervention ranged from 7 days to 20 weeks. Among the 42 studies, 16 (38.1%) conducted interventions for less than 8 weeks, while 26 studies (61.9%) applied interventions for a longer term, that is, 8 weeks or more (Figure 3D). Detailed information on the resveratrol used in each study is shown in Table 3.

**TABLE 3:** Information on the resveratrol used in each study.

Study, year	Source	Purity (%)	Quality control reported
Sharma et al., 2006	Sigma–Aldrich, St. Louis, MO, USA	≥99%	HPLC
Ding et al., 2010	Sigma–Aldrich, St. Louis, MO, USA	≥99%	HPLC
Chang et al., 2011	Sigma–Aldrich, St. Louis, MO, USA	≥99%	HPLC
Chen et al., 2011	Sigma–Aldrich, St. Louis, MO, USA	≥99%	HPLC
Palsamy et al., 2011	Sigma–Aldrich, St. Louis, MO, USA	≥99%	HPLC
Khamneh et al., 2012	Cayman chem., Ann Arbor, MI, USA	Unknown	Unknown
Ramar et al., 2012	Sigma–Aldrich, St. Louis, MO, USA	≥99%	HPLC
Wu et al., 2012	Unknown	Unknown	Unknown
Huang et al., 2013	Zelang, Nanjing, China	>98%	HPLC
Kim et al., 2013	Sigma–Aldrich, St. Louis, MO, USA	≥99%	HPLC
Jiang et al., 2013	JF‐NATURAL, Tianjin, China	>98%	Unknown
Wen et al., 2013	Copalyton Chemical Materials Co., Ltd, Shanghai, China	Unknown	Unknown
Xu et al., 2014	Unknown	Unknown	Unknown
Zhou et al., 2014	Zhuangtai Company, Nanjing, China	Unknown	Unknown
Yaylali et al., 2015	Sigma–Aldrich, St. Louis, MO, USA	≥99%	HPLC
Elbe et al., 2015	Molekula, UK	Unknown	Unknown
He et al., 2016	Sigma–Aldrich, St. Louis, MO, USA	≥99%	HPLC
Hussein et al., 2016	Sigma–Aldrich, St. Louis, MO, USA	≥99%	HPLC
Wang, et al., 2016	Sigma–Aldrich, St. Louis, MO, USA	≥99%	HPLC
Park et al., 2016	Sigma–Aldrich, St. Louis, MO, USA	≥99%	HPLC
Yan et al., 2016	Sigma–Aldrich, St. Louis, MO, USA	≥99%	HPLC
Qiao et al., 2017	Cayman chem., Ann Arbor, MI, USA	unknown	unknown
Xu et al., 2017	Sigma–Aldrich, St. Louis, MO, USA	≥99%	HPLC
Huang et al., 2017	Sigma–Aldrich, St. Louis, MO, USA	≥99%	HPLC
Yuan et al.,2018	Sigma–Aldrich, St. Louis, MO, USA	≥99%	HPLC
Bashir., 2018	Sigma–Aldrich, St. Louis, MO, USA	≥99%	HPLC
Maity et al., 2018	Sigma–Aldrich, St. Louis, MO, USA	≥99%	HPLC
Rehman et al., 2018	Bristol Mayer Biotech Pakistan	unknown	unknown
Zhang et al., 2019	Sigma–Aldrich, St. Louis, MO, USA	≥99%	HPLC
Peng et al., 2019	Sigma–Aldrich, St. Louis, MO, USA	≥99%	HPLC
Xian et al., 2019	Unknown	Unknown	Unknown
Xian et al., 2020	Sigma–Aldrich; Merck KGaA	≥99%	HPLC
Du et al., 2019	Sigma–Aldrich, St. Louis, MO, USA	≥99%	HPLC
Du et al., 2020	MedChemExpress, Shanghai, China	99.94%	HPLC
Cai et al., 2020	Unknown	Unknown	Unknown
Szkudelska et al., 2020	Sigma–Aldrich, St. Louis, MO, USA	≥99%	HPLC
Wang et al., 2020	Sigma–Aldrich, St. Louis, MO, USA	≥99%	HPLC
Zhu et al., 2020	Sigma–Aldrich, St. Louis, MO, USA	≥99%	HPLC
Zhang et al., 2020	Sigma–Aldrich, St. Louis, MO, USA	≥99%	HPLC
Qi et al., 2021	Unknown	Unknown	Unknown
Gu et al., 2021	Sigma–Aldrich, St. Louis, MO, USA	≥99%	HPLC
Zhang et al., 2022	MedChemExpress, Shanghai, China	99.94%	HPLC

Abbreviation: HPLC, high‐performance liquid chromatography.

### 3.3 Study quality

**FIGURE 4:**
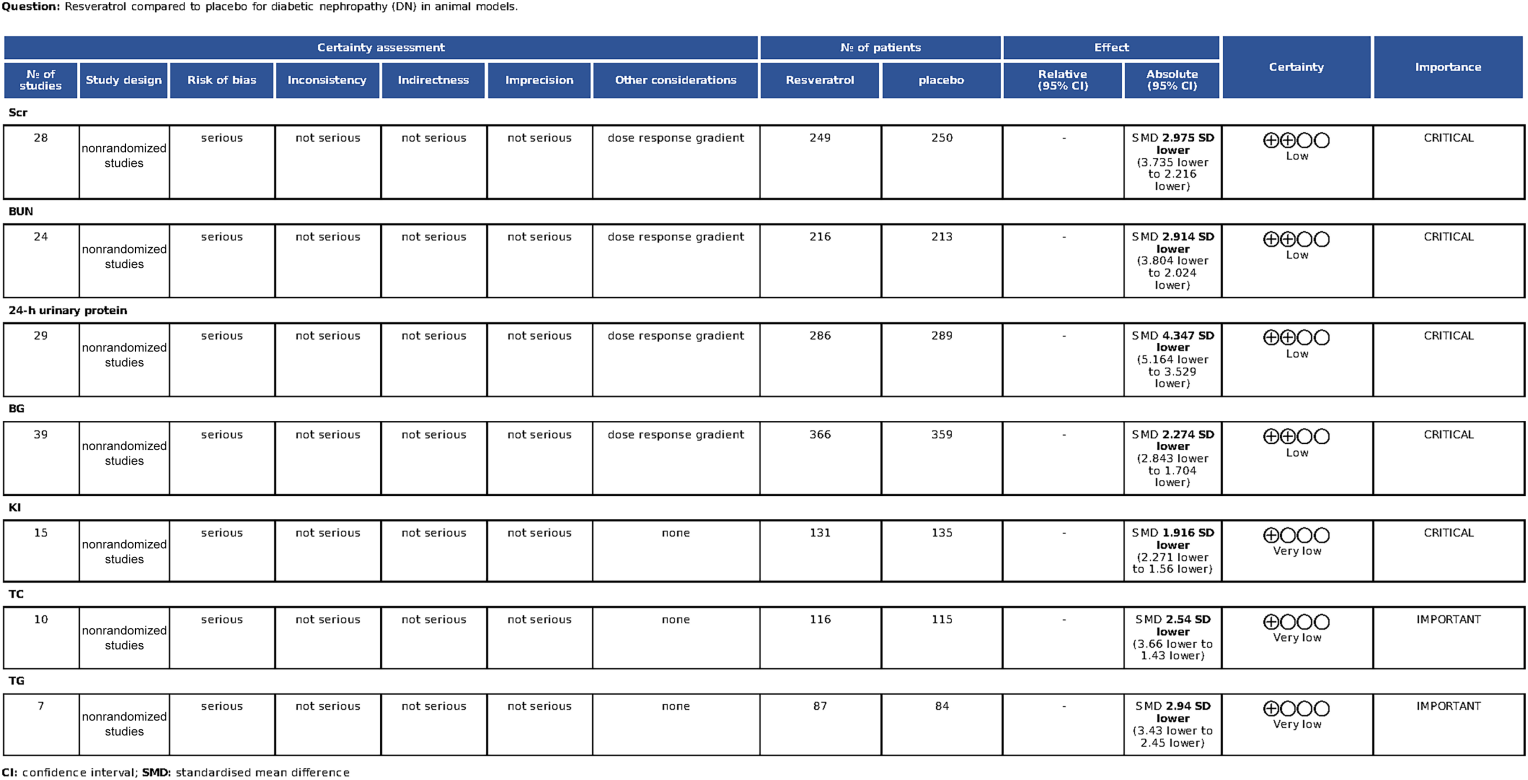
GRADE evidence summary table.

Random allocation to experimental and control groups was mentioned in 29 studies (69.0%), and the remaining 13 studies did not report the methods of allocation. Eight studies reported whether the distribution of relevant baseline levels was balanced between the experimental groups and control groups. Random housing, blinding (performance bias), and random outcome assessment were not mentioned in any of the studies. All these studies had complete outcome data and reported expected outcomes. For other sources of bias, 38 studies (90.5%) stated that there was no conflict of interest among the authors, and the remaining four studies (9.5%) did not mention it. The methodological quality of the included studies is displayed in Table 4. The quality assessment of the evidence is outlined in Figure 4.

**TABLE 4:** Risk of bias of included studies.

Study (year)	(1)	(2)	(3)	(4)	(5)	(6)	(7)	(8)	(9)	(10)
Sharma et al. (2006)	Y	U	U	U	U	U	Y	Y	Y	Y
Ding et al. (2010)	U	U	U	U	U	Y	Y	Y	Y	U
Chang et al. (2011)	Y	U	U	U	U	U	Y	Y	Y	Y
Chen et al. (2011)	U	U	U	U	U	U	Y	Y	Y	U
Palsamy, et al. (2011)	U	U	U	U	U	U	Y	Y	Y	U
Khamneh et al. (2012)	Y	U	U	U	U	U	Y	Y	Y	Y
Ramar et al. (2012)	U	U	U	U	U	U	Y	Y	Y	U
Wu et al. (2012)	U	U	U	U	U	U	Y	Y	Y	U
Jiang et al. (2013)	Y	U	U	U	U	U	Y	Y	Y	Y
Kim et al. (2013)	U	Y	U	U	U	U	Y	Y	Y	Y
Wen et al. (2013)	Y	U	U	U	U	U	Y	Y	Y	Y
Huang et al. (2013)	Y	U	U	U	U	U	Y	Y	Y	Y
Xu et al. (2014)	Y	U	U	U	U	U	Y	Y	Y	Y
Elbe et al. (2014)	Y	Y	U	U	U	U	Y	Y	Y	Y
Zhou et al. (2014)	Y	U	U	U	U	U	Y	Y	Y	Y
Yaylali et al. (2015)	Y	Y	U	U	U	U	Y	Y	Y	Y
He et al. (2016)	Y	U	U	U	U	U	Y	Y	Y	Y
Hussein et al. (2016)	U	U	U	U	U	U	Y	Y	Y	Y
Park et al. (2016)	U	Y	U	U	U	U	Y	Y	Y	Y
Wang, et al. (2016)	Y	U	U	U	U	U	Y	Y	Y	Y
Yan et al. (2016)	U	Y	U	U	U	U	Y	Y	Y	Y
Xu et al. (2017)	Y	U	U	U	U	U	Y	Y	Y	Y
Huang et al. (2017)	U	U	U	U	U	U	Y	Y	Y	Y
Qiao et al. (2017)	U	Y	U	U	U	U	Y	Y	Y	Y
Yuan et al. (2018)	Y	U	U	U	U	U	Y	Y	Y	Y
Peng et al. (2018)	U	U	U	U	U	U	Y	Y	Y	Y
Bashir et al. (2018)	Y	U	U	U	U	U	Y	Y	Y	Y
Maity et al. (2018)	Y	U	U	U	U	U	Y	Y	Y	Y
Zhang et al. (2018)	Y	U	U	U	U	U	Y	Y	Y	Y
Rehman et al. (2018)	U	U	U	U	U	U	Y	Y	Y	Y
Xian et al. (2019)	Y	U	U	U	U	U	Y	Y	Y	Y
Du et al. (2019)	Y	Y	U	U	U	Y	Y	Y	Y	Y
Xian et al. (2019)	Y	U	U	U	U	U	Y	Y	Y	Y
Zhu et al. (2020)	U	U	U	U	U	U	Y	Y	Y	U
Du et al. (2020)	Y	Y	U	U	U	Y	Y	Y	Y	Y
Cai et al. (2020)	Y	U	U	U	U	Y	Y	Y	Y	Y
Zhang et al. (2020)	Y	U	U	U	U	U	Y	Y	Y	Y
Szkudelska et al. (2020)	Y	U	U	U	U	U	Y	Y	Y	Y
Wang et al. (2020)	Y	U	U	U	U	Y	Y	Y	Y	Y
Qi et al. (2021)	Y	U	U	U	U	U	Y	Y	Y	Y
Gu et al. (2021)	U	U	U	U	U	U	Y	Y	Y	Y
Zhang et al. (2022)	Y	U	U	U	U	Y	Y	Y	Y	Y

(1) Sequence generation, (2) baseline characteristics, (3) allocation concealment, (4) random housing, (5) blinding (performance bias), (6) random outcome assessment, (7) blinding (detection bias), (8) incomplete outcome data, (9) selective outcome reporting, and (10) other sources of bias.

Abbreviations: N, no; U, unclear; Y, yes.

### 3.4 Effectiveness

#### 3.4.1 Golden standard and morphometric parameters

Renal pathology is the gold standard for treating DN. Histopathological analysis of renal tissue revealed a decrease in the thickness of the glomerular basement membrane (GBM) in three studies, a decrease in the glomerular diameter in six studies, an increase in the glomerular sclerosis index in four studies, and significant effects of resveratrol on ameliorating glomerular mesangial cell proliferation in DN animals in five studies, as assessed using light and electron microscopy, compared with those in the model group.

**FIGURE 5:**
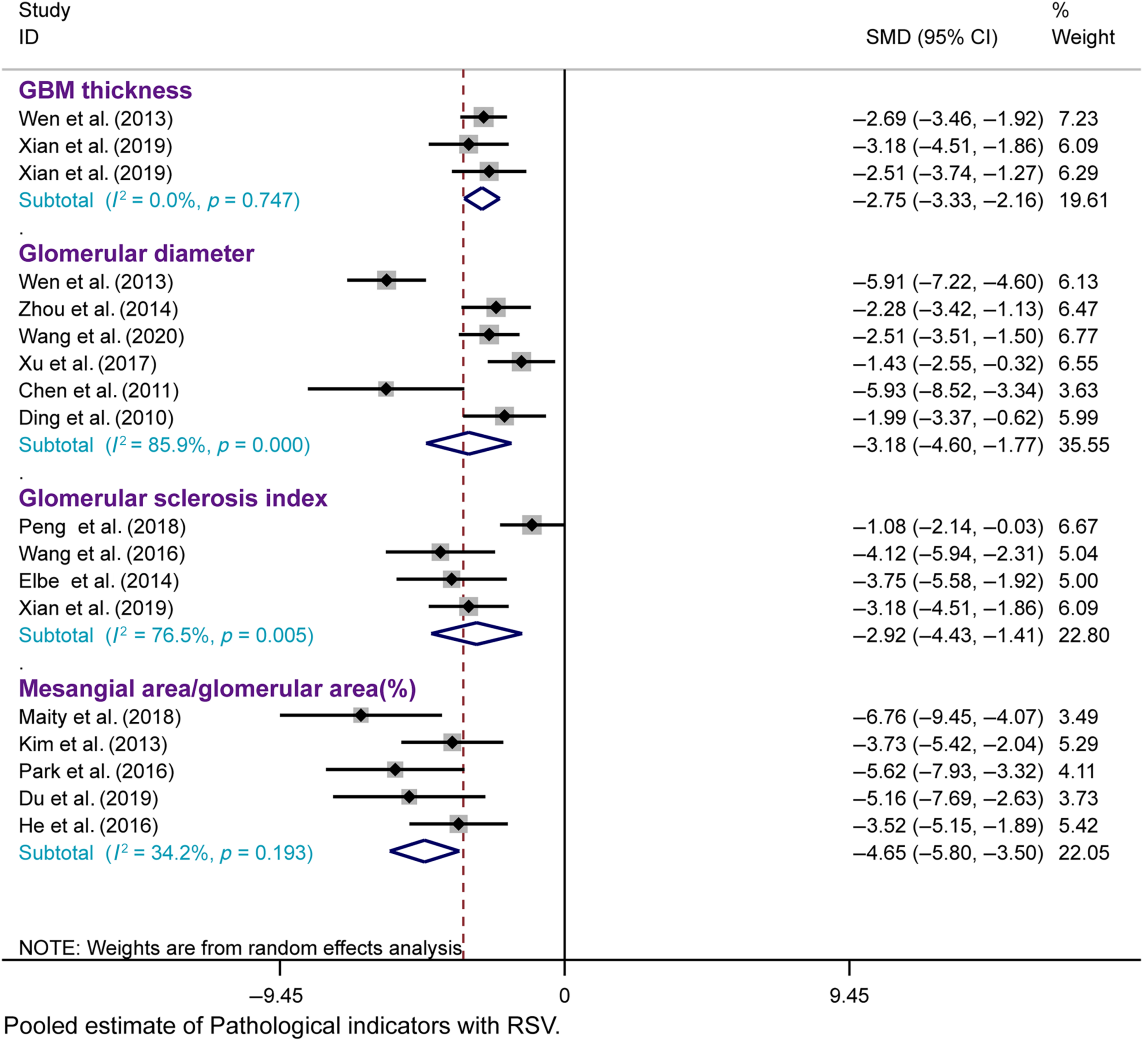
Forest plot: Effect of resveratrol on pathological indicators.

Three studies were included, and a fixed effects model was used. Resveratrol treatment of the DN significantly decreased the GBM thickness (*n* = 90, SMD = −2.746, 95% CI [−3.333, −2.158], *p* = 0.000 < 0.01; heterogeneity: χ^2^ = 0.58, *I* ^2^ = 0.0%). A total of six studies were included, and a random effects model was used. Resveratrol treatment of the DN significantly decreased the glomerular diameter (*n* = 141, SMD = −3.183, 95% CI [−4.599, −1.767], *p* = 0.000 < 0.01; heterogeneity: χ^2^ = 35.59, *I* ^2^ = 85.9%). A total of four studies were included, and a random effects model was used. Retreatment of the DN significantly decreased the glomerular diameter (*n* = 67, SMD = −2.921, 95% CI [−4.429, −1.414], *p* = 0.000 < 0.01; heterogeneity: χ^2^ = 12.76, *I* ^2^ = 76.5%). The degree of renal injury, such as mesangial matrix deposition, was always quantitatively estimated by the mesangial area ratio, which was calculated as the mesangial area/glomerular area. A total of five studies were included, and a fixed effects model was used. Resveratrol treatment of the DN significantly reduced the mesangial area ratio (*n* = 96, SMD = −4.651, 95% CI [−5.803, −3.498], *p* = 0.000 < 0.01; heterogeneity: χ^2^ = 6.08, *I* ^2^ = 34.2%) (Figure 5). These findings show that resveratrol may be effective at alleviating renal pathology and impairment in DN animal models.

#### 3.4.2 Kidney functional parameters

##### Scr, BUN, and CCr

**FIGURE 6:**
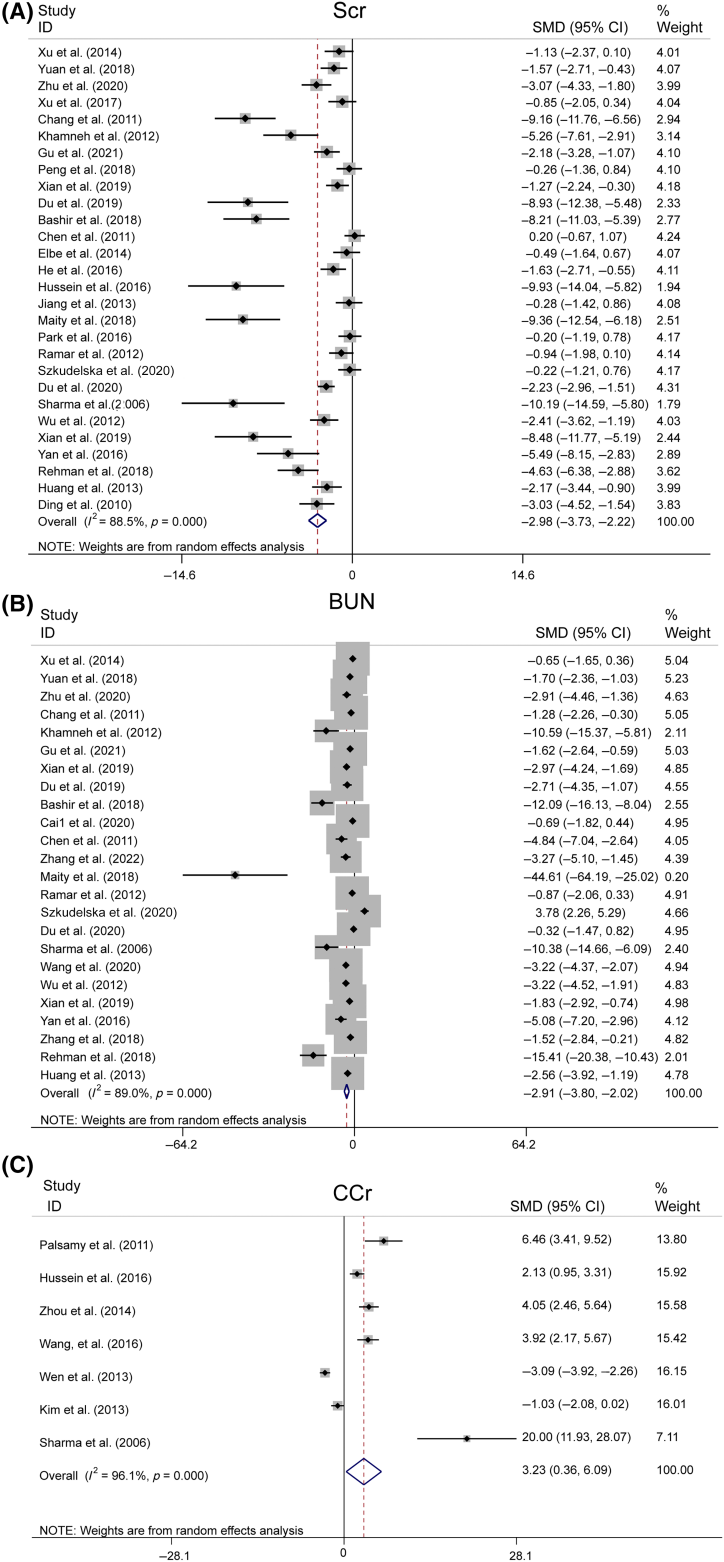
Forest plot: Effect of resveratrol on kidney functional parameters. (A) Overall effect of resveratrol on Scr. (B) Overall effect of resveratrol on BUN. (C) Overall effect of resveratrol on CCr.

Our meta‐analysis of 28 studies revealed significantly lower Scr levels in the resveratrol group than in the model group (*n* = 499, SMD = −2.975, 95% CI [−3.735, −2.216], *p* = 0.000 < 0.01; heterogeneity: χ^2^ = 235.42, *I* ^2^ = 88.5%, Figure 6A). Egger's test for publication bias in Scr (|*t*| = 0.000 < 0.05) suggested its presence (Figure S1A). To address funnel plot asymmetry due to publication bias, we employed a trim‐and‐fill analysis. This method confirmed the consistency of the outcomes, validating the robustness of our findings (Figure S1B).

Our meta‐analysis, encompassing 24 studies, showed that BUN levels were significantly lower in the resveratrol group than in the model group (*n* = 429, SMD = −2.914, 95% CI [−3.804, −2.024], *p* < 0.01; heterogeneity: χ^2^ = 208.45, *I* ^2^ = 89.0%, Figure 6B). However, Egger's test suggested potential publication bias for BUN (|*t*| = 0.00 > 0.05, Figure S1C). After applying the trim‐and‐fill method to correct this, the results were consistent, confirming the reliability of the findings (Figure S1D).

Sensitivity analyses for Scr and BUN demonstrated that excluding any single study did not notably reduce heterogeneity (Figure S2A,B), thus reinforcing the reliability of the initial findings. However, an analysis of seven studies^24^, ^34^, ^38^, ^41^, ^50^, ^51^, ^57^ indicated that resveratrol did not significantly modulate CCr compared to that in the model group (*n* = 146, SMD = 3.225, 95% CI [0.364, 6.087], *p* = 0.027 > 0.01; heterogeneity: χ^2^ = 154.47, *I* ^2^ = 96.1%, Figure 6C).

##### Levels of 24‐h urinary protein, urine volume, and Alb

**FIGURE 7:**
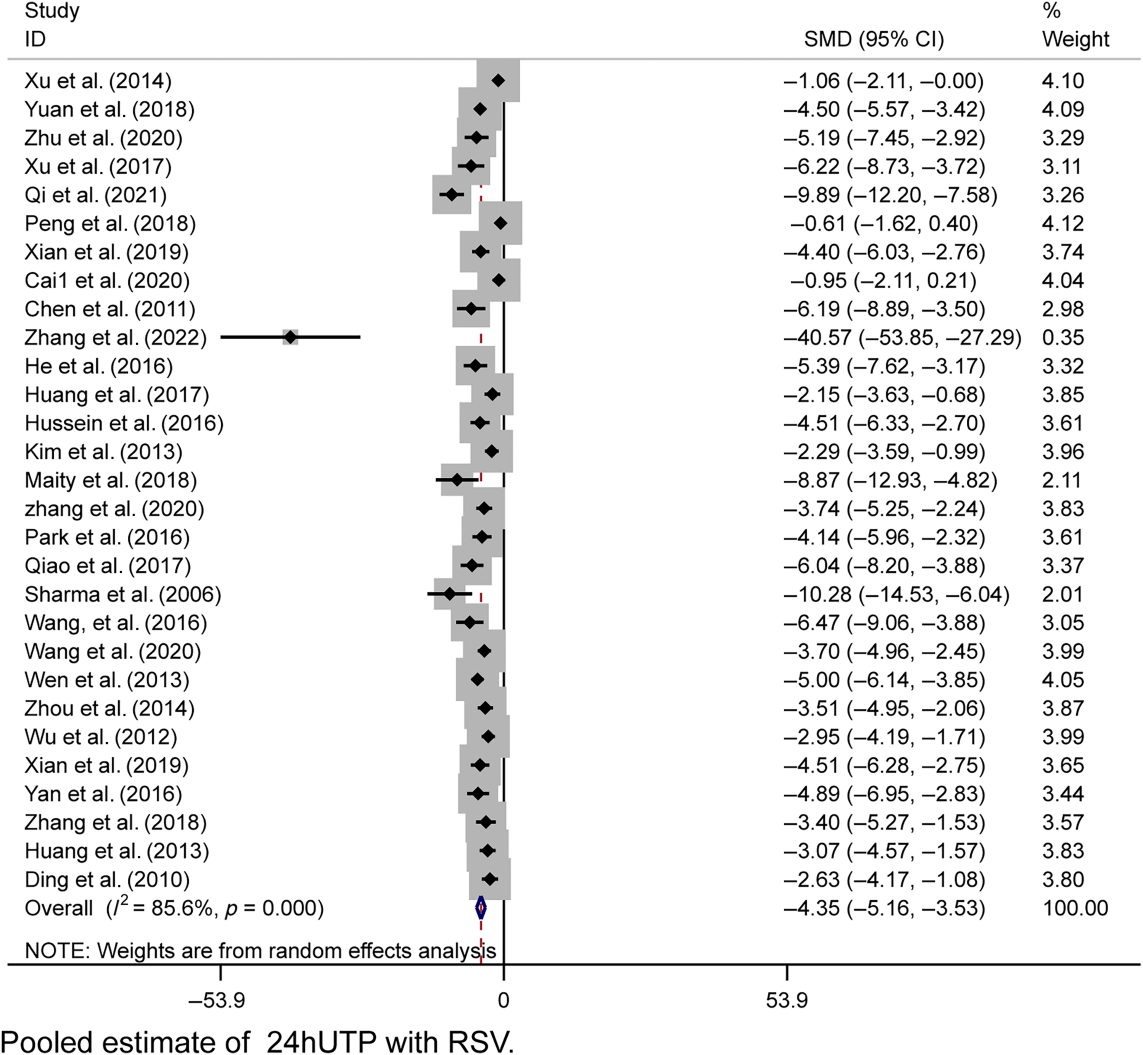
Forest plot: Effect of resveratrol on 24hUTP.

In the pooled analysis, proteinuria was evaluated across various metrics, including the 24‐h urinary albumin excretion rate, total 24‐h urinary albumin, the urine albumin‐to‐creatinine ratio (UACR), and 24‐h urinary microalbumin. Collectively, these results were referred to as 24hUTP. A random‐effects model was used for the analysis of 29 studies, revealing significantly lower 24hUTP levels in the resveratrol group (*n* = 575, SMD = −4.347, 95% CI [−5.164, −3.529], *p* < 0.01; heterogeneity: χ^2^ = 194.96, *I* ^2^ = 85.6%, Figure 7). Egger's test suggested publication bias for 24hUTP (|*t*| = 0.000 < 0.05, Figure S1E), which was addressed using the trim‐and‐fill method. This procedure confirmed the stability of our results, with no change in the relational outcomes (Figure S1F). Sensitivity analyses for 24hUTP showed no significant decrease in heterogeneity upon the exclusion of individual studies (Figure S2C).

The meta‐analysis of six studies revealed that urine volume was markedly lower in the resveratrol group than in the model group (*n* = 98, SMD = −7.755, 95% CI [−11.447, −4.062], *p* < 0.01; heterogeneity: χ^2^ = 68.12, *I* ^2^ = 92.7%, Figure S3A). Analysis of two studies revealed that resveratrol did not significantly increase Alb levels (Figure S3B). Resveratrol significantly improved kidney function, as evidenced by reduced levels of Scr, BUN, 24‐h UTP, and urine output (*p* < 0.01).

#### 3.4.3 Metabolic parameters and biochemical parameters

##### Levels of BG and HbA1c

**FIGURE 8:**
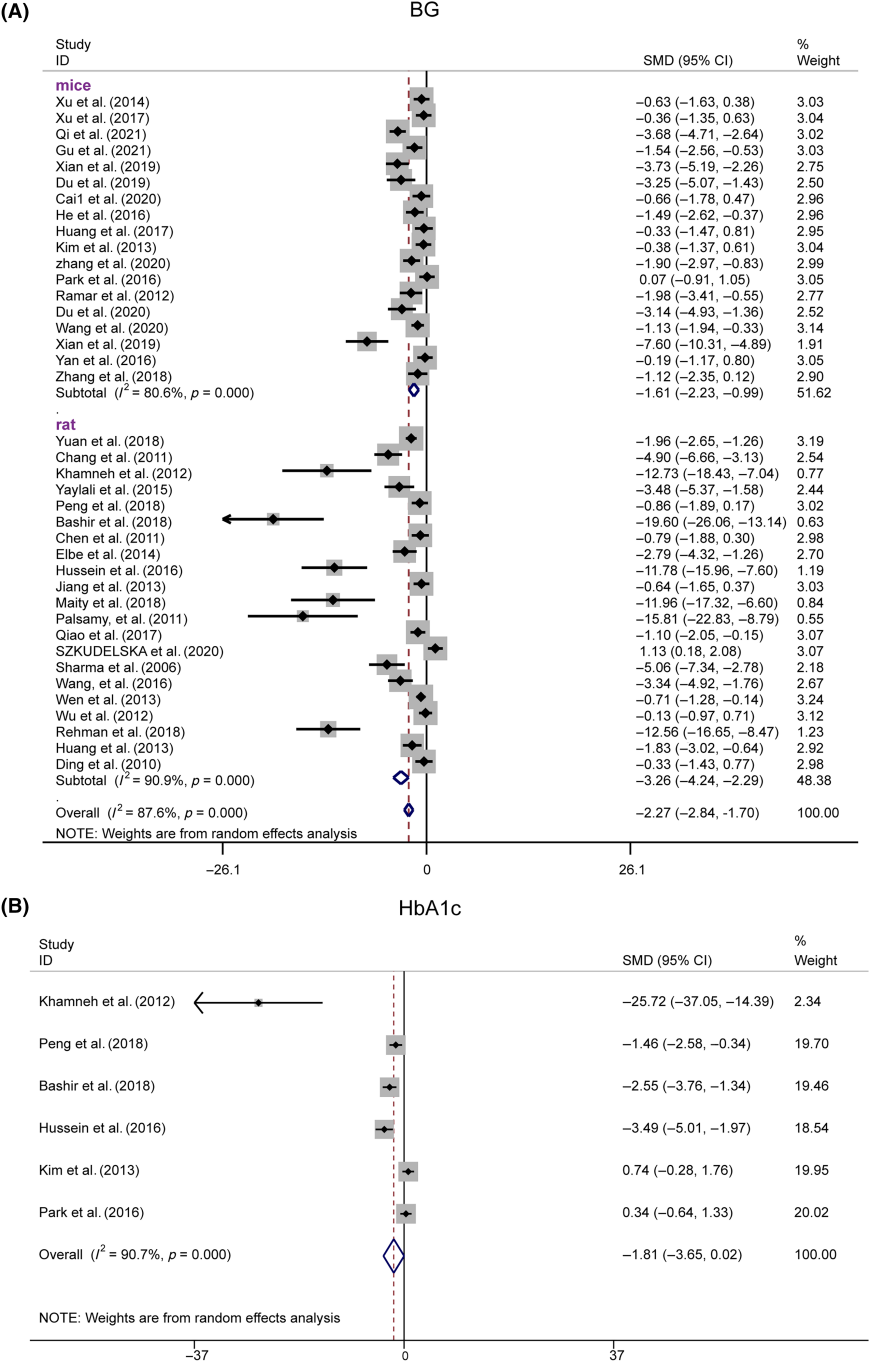
Forest plot: Effect of resveratrol on blood sugar indicators. (A) The overall effect of resveratrol on BG. (B) The overall effect of resveratrol on HbA1c.

Thirty‐nine studies reported significantly lower BG levels in the resveratrol group (*n* = 725, SMD = −2.274, 95% CI [−2.843, −1.704], *p* < 0.01; heterogeneity: χ^2^ = 307.61, *I* ^2^ = 87.6%, Figure 8A). Sensitivity analyses revealed no significant decrease in heterogeneity after excluding any studies (Figure S2D). Egger's test indicated publication bias for BG (|*t*| = 0.000 < 0.05, Figure S1G), which was corrected using trim and fill, with the p value remaining at 0.000 (Figure S1H).

Six studies evaluated HbA1c levels postresveratrol treatment. A plasma HbA1c of 0.7 showed significant heterogeneity (*I* ^2^ = 90.7%), suggesting the use of a random‐effects model for analysis. The HbA1c levels were not significantly different from those in the model group (*n* = 98, SMD = −1.814, 95% CI [−3.649, 0.022], *p* = 0.053 > 0.01; heterogeneity: χ^2^ = 53.74, *I* ^2^ = 90.7%, Figure 8B). Stratified analysis by species revealed significant BG improvements in both the rat and mouse subgroups, whereas HbA1c showed significant effects in the rats but not in the mice.

##### BW, KW, KI, and SBP

Considering the variability in BW measurements, we meta‐analyzed kidney indices and found that resveratrol significantly reduced KW and KI, with no changes in BW or SBP (Figure S5A,C). Thirteen studies reported KW with resveratrol, showing significant heterogeneity (*I* ^2^ = 87.9%), suggesting the use of a random effects model. KW did not differ significantly from that of the controls (*n* = 240, SMD = −1.679, 95% CI [−2.637, −0.720], *p* = 0.001; χ^2^ = 98.92, *I* ^2^ = 87.9%, Figure S5B). Egger's test showed no publication bias (*p* > |*t*| = 0.135, Figure S1I).

**FIGURE 9:**
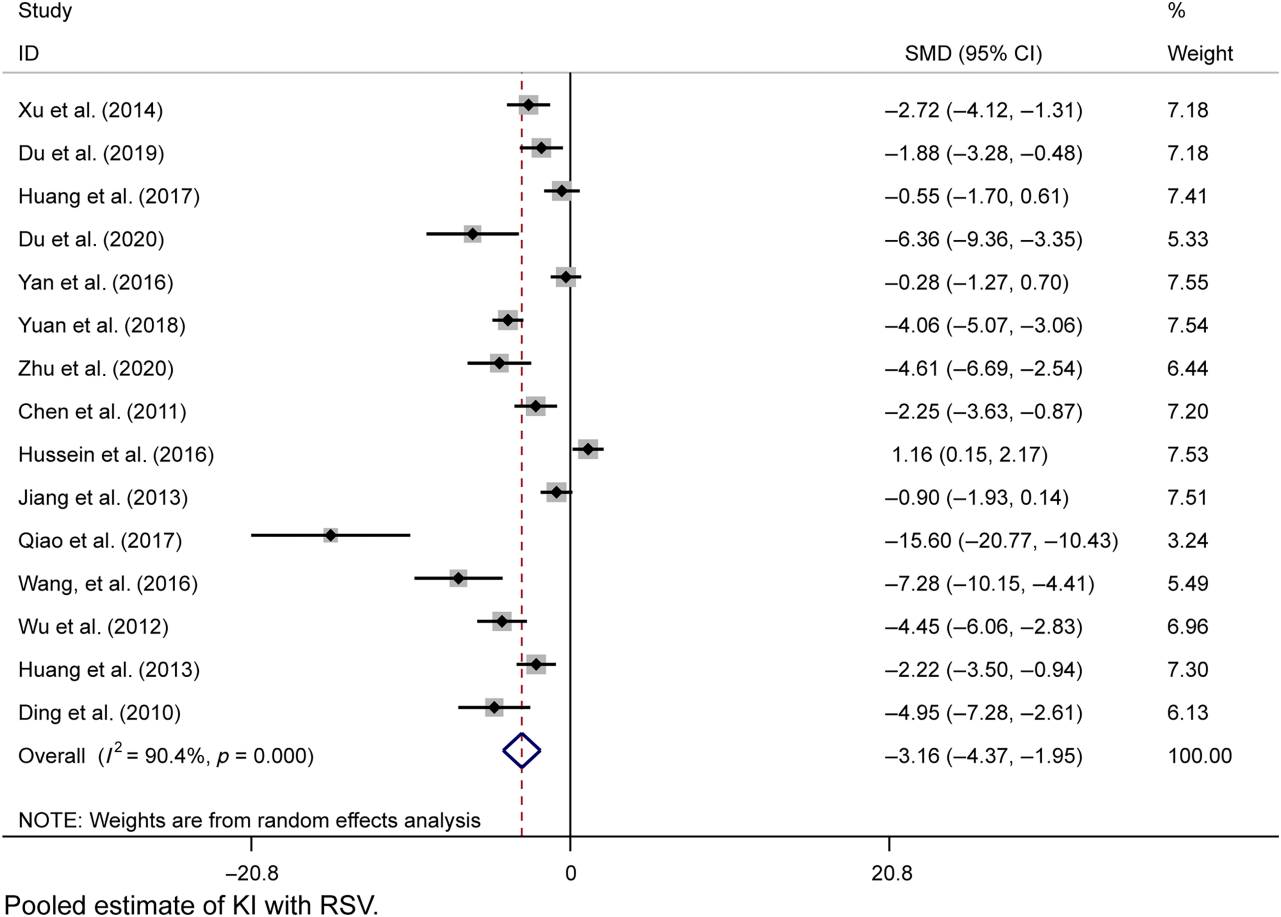
Forest plot: Effect of resveratrol on KI.

Fifteen studies provided postresveratrol KI data with considerable heterogeneity (*I* ^2^ = 90.1%), and a random effects model was used. KI was significantly different from the model group (*n* = 266, SMD = −1.916, 95% CI [−2.271, −1.560], *p* = 0.000; χ^2^ = 145.79, *I* ^2^ = 90.4%, Figure 9). Egger's test indicated potential publication bias (*p* > |*t*| = 0.001, Figure S1J), but trim‐and‐fill analysis confirmed the results (Figure S1K). Sensitivity analyses were within the 95% CI (Figure S2E,F).

##### TC, TG, LDL‐C, and HDL‐C

Uncontrolled serum lipid profiles, key contributors to DN, were evaluated in a meta‐analysis of all included studies. Resveratrol significantly lowered TC, TG, and LDL‐C levels (Figure S5A–C), with no notable effect on HDL‐C (*p* = 0.088, Figure S5D). Sensitivity analysis confirmed the robustness of the TC, TG, and LDL‐C results, as all estimates were within the 95% CI (Figure S2G).

### 3.5 Heterogeneity and results of subgroup analysis

Potential confounders such as diverse modeling methods, rodent species, resveratrol dosing (daily/total), and treatment duration may contribute to outcome heterogeneity. In our systematic review and meta‐analysis, we explored this heterogeneity through meta‐regression and subgroup analyses, with specific data detailed in Tables 5, 6, S1, and S2.

**TABLE 5:** Stratified analysis of pooled estimates according to Scr.

Variables	Experiments (*n*)	Individuals (*n*)	SMD	95% CI	*p* value	Heterogeneity
Single dose						
0–20	7	94	−6.010	(−9.075, −2.944)	*p* = 0.000 < 0.01	χ^2^ = 88.28, *I* ^2^ = 93.2%
20–100	16	313	−2.162	(−3.015, −1.309)	*p* = 0.000 < 0.01	χ^2^ = 117.22, *I* ^2^ = 87.2%
100–200	5	92	−2.621	(−3.887, −1.356)	*p* = 0.000 < 0.01	χ^2^ = 18.15, *I* ^2^ = 78.0%
Total dose						
0–100	7	101	−4.377	(−7.017, −1.737)	*p* = 0.001 < 0.01	χ^2^ = 63.67, *I* ^2^ = 92.1%
100–500	16	306	−3.384	(−4.906, −1.862)	*p* = 0.000 < 0.01	χ^2^ = 126.28, *I* ^2^ = 92.9%
>500	5	92	−2.238	(−2.964, −1.512)	*p* = 0.000 < 0.01	χ^2^ = 40.45, *I* ^2^ = 72.8%
Period						
≤8 W	17	308	−2.557	(−3.438, −1.676)	*p* = 0.000 < 0.01	χ^2^ = 124.70, *I* ^2^ = 87.2%
>8 W	11	191	−3.735	(−5.228, −2.243)	*p* = 0.000 < 0.01	χ^2^ = 108.94, *I* ^2^ = 90.8%
Species						
Rats	17	292	−3.517	(−4.703, −2.331)	*p* = 0.000 < 0.01	χ^2^ = 177.23, *I* ^2^ = 91.0%
Mice	11	207	−2.240	(−3.149, −1.331)	*p* = 0.000 < 0.01	χ^2^ = 58.10, *I* ^2^ = 82.8%
Modeling methods						
Genetic background	22	405	−2.307	(−3.738, −0.876)	*p* = 0.002 < 0.01	χ^2^ = 33.34, *I* ^2^ = 85.0%
STZ/Alloxan	6	94	−3.182	(−4.086, −2.278)	*p* = 0.000 < 0.01	χ^2^ = 199.70, *I* ^2^ = 89.5%
Overall	28	499	−2.975	(−3.735, −2.216)	*p* = 0.000 < 0.01	χ^2^ = 235.42, *I* ^2^ = 88.5%

*Note*: See Supporting Information Appendix S2 for abbreviations. *p* < 0.05 represents a significant difference.

**TABLE 6:** Stratified analysis of pooled estimates according to 24hUTP.

Variables	Experiments (*n*)	Individuals (*n*)	SMD	95% CI	*p* value	Heterogeneity
Single dose						
0–20	10	156	−3.361	(−4.687, −2.035)	*p* = 0.000 < 0.01	χ^2^ = 63.39, *I* ^2^ = 85.8%
20–100	15	323	−4.359	(−5.231, −3.487)	*p* = 0.000 < 0.01	χ^2^ = 54.91, *I* ^2^ = 74.5%
100–200	4	96	−6.158	(−8.551, −3.765)	*p* = 0.000 < 0.01	χ^2^ = 16.99, *I* ^2^ = 82.3%
Total dose						
0–100	8	123	−4.780	(−6.725, −2.834)	*p* = 0.001 < 0.01	χ^2^ = 56.97, *I* ^2^ = 87.7%
100–500	16	336	−3.779	(−4.762, −2.796)	*p* = 0.000 < 0.01	χ^2^ = 96.87, *I* ^2^ = 84.5%
>500	5	116	−5.620	(−7.575, −3.664)	*p* = 0.000 < 0.01	χ^2^ = 21.56, *I* ^2^ = 81.4%
Period						
≤8 W	15	344	−5.096	(−6.421, −3.770)	*p* = 0.000 < 0.01	χ^2^ = 122.72, *I* ^2^ = 88.6%
>8 W	14	231	−3.614	(−4.551, −2.677)	*p* = 0.000 < 0.01	χ^2^ = 60.09, *I* ^2^ = 78.4%
Species						
Rats	15	314	−4.897	(−6.161, −3.634)	*p* = 0.000 < 0.01	χ^2^ = 107.06, *I* ^2^ = 86.9%
Mice	14	261	−3.890	(−4.974, −2.806)	*p* = 0.000 < 0.01	χ^2^ = 60.09, *I* ^2^ = 78.4%
Overall	29	575	−4.347	(−5.160, −3.529)	*p* = 0.000 < 0.01	χ^2^ = 194.96, *I* ^2^ = 85.6%

*Note*: See Supporting Information Appendix S2 for abbreviations. *p* < 0.05 represents a significant difference.

For the Scr outcome, meta‐regression identified total and single doses, treatment duration, species, and disease model as sources of heterogeneity (Table 5). Subgrouping by daily and total resveratrol dosages revealed reduced heterogeneity in the low‐ and high‐dosage subgroups, with moderate dosages still showing high heterogeneity. There was a slight decrease in heterogeneity for both short‐ and long‐duration treatments. Mouse models showed significant heterogeneity reduction, whereas rat models did not. Subgroup analysis by model method notably decreased heterogeneity in mutant mice, with no significant change in the STZ/Alloxan injection group.

For the BUN outcome, meta‐regression analysis identified total and single doses and species as contributors to heterogeneity (Table S1). The treatment duration and model methods did not significantly contribute (*p* > |*z*| for duration = 0.05, for model methods = 0.659). Subgroup analysis by species revealed a significant reduction in heterogeneity in the mouse subgroup. High total/single doses led to a substantial decrease in heterogeneity, whereas low doses resulted in a mild decrease. A moderate reduction in heterogeneity was observed in the mutant mouse group based on the model methods, although this difference was not statistically significant.

For the 24hUTP outcome, meta‐regression showed that total and single doses, duration, and species contributed to heterogeneity, whereas model methods did not. Sensitivity analysis revealed no outlier studies influencing heterogeneity. Furthermore, total dose, daily dose, duration, and species were significant sources of heterogeneity (*p* = 0.000 for each), whereas the model did not (*p* > |*z*| = 0.068). Subgroup analysis by rodent species, resveratrol dose, and treatment duration provided more insightful results (Table 6). Notably, heterogeneity was significantly reduced in subgroups with single doses greater than 100 mg, total doses greater than 500 mg, and treatment durations exceeding 8 weeks, particularly in studies involving mice.

For the BG outcome, meta‐regression analysis (Table S2) identified total and single doses, duration, and species as sources of heterogeneity. Model methods did not significantly contribute (*p* > |*z*| = 0.263). Notably, heterogeneity was substantially reduced in subgroups with single doses above 100 mg and total doses above 500 mg.

Heterogeneity in study outcomes, as shown by meta‐regression and subgroup analyses, can be ascribed to factors such as variability in modeling methods, rodent species, resveratrol dosing (daily and total), and treatment duration. These factors significantly influenced the results, indicating that the effects of resveratrol may vary and that certain dosages and durations could be more effective.

Reduced heterogeneity in subgroups with low and high resveratrol dosages and in those with treatments over 8 weeks suggested dose‐ and time‐dependent effects on outcomes such as Scr, BUN, and 24hUTP. This trend was especially clear in mouse studies, where higher doses and longer durations led to more consistent and significant results. These findings warrant a dose‐dependent analysis to determine the optimal resveratrol dosage and treatment duration for therapeutic effects, clarifying the dosage thresholds for maximum efficacy.

### 3.6 Proposed renoprotective mechanisms

The renoprotective mechanisms of resveratrol against DN are complex, multifaceted, and involve multiple targets, as detailed in Table S3.

### 3.7 Effective dose of resveratrol

**FIGURE 10:**
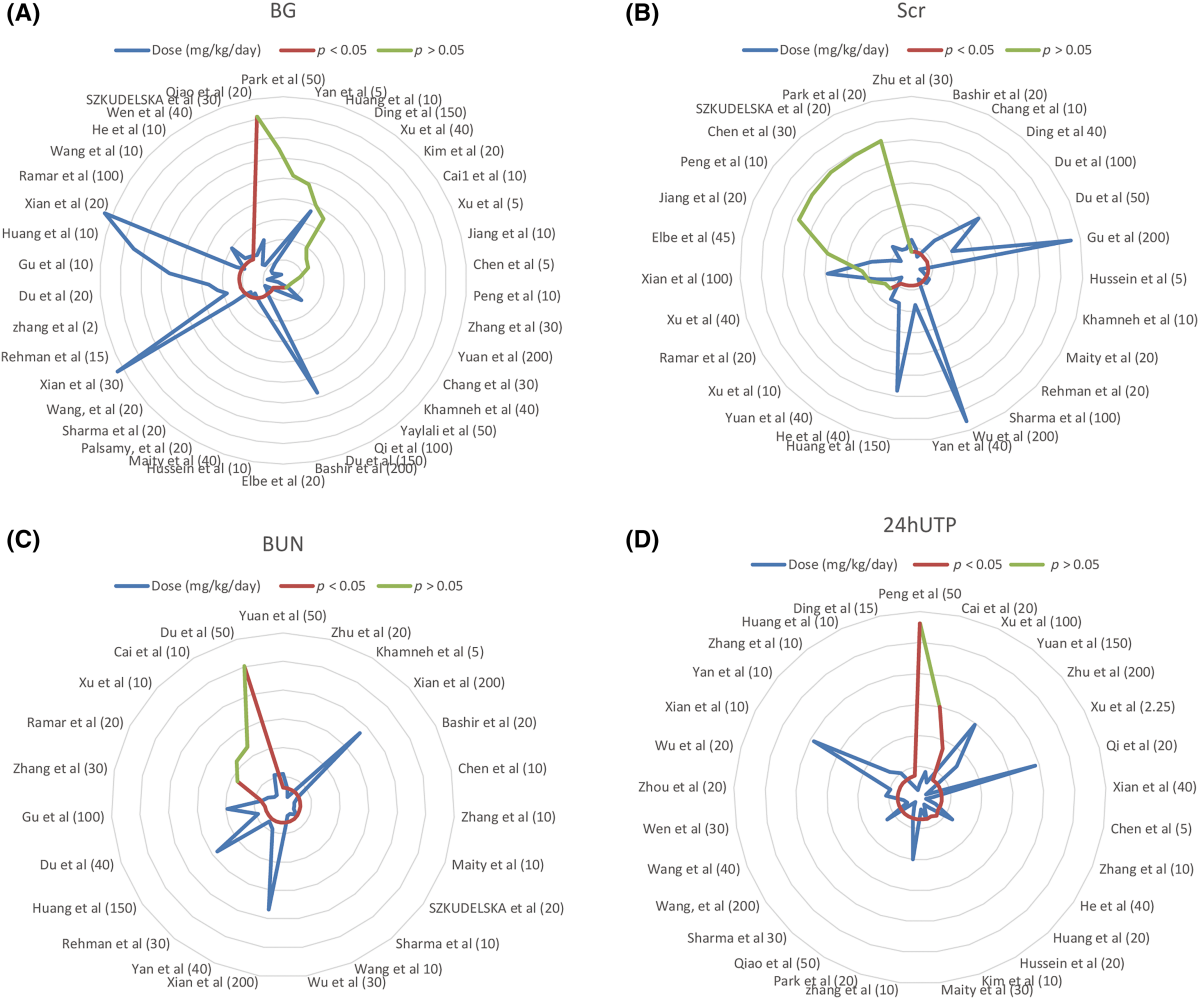
Dose–response radar maps of (A) resveratrol therapy and BG, (B) resveratrol therapy and Scr, (C) resveratrol therapy and BUN, and (D) resveratrol therapy and 24hUTP.

The dose–response relationship is pivotal for clinical medication, and to ascertain the optimal resveratrol dosage, we developed a dose–response radar map. Resveratrol effectively reduced BG levels at both low (2–5 mg/kg/day, *n* = 4) and high dosages (100–200 mg/kg/day, *n* = 6), with 83.3% efficacy observed in the higher range. However, a moderate dosage of 5–100 mg/kg/day (*n* = 28) showed a 71.4% effectiveness rate (*n* = 20), and 28.6% (*n* = 8) were ineffective, highlighting more pronounced effects at the lower and higher dosages (Figure 10A). For Scr, a similar pattern emerged, with resveratrol at dosages of ≥5–100 mg/kg/day or 2–5 mg/kg/day (*n* = 6, 83.3%) demonstrating significant effects (Figure 10B). Compared to the 15–50 mg/kg/day dose (*n* = 15, 86.7%), both the low (≤15 mg/kg/day, *n* = 10, 100%) and high (100–200 mg/kg/day, *n* = 4, 100%) resveratrol doses significantly lowered the BUN concentration (Figure 10C). Furthermore, higher daily doses (100–200 mg/kg) of the model group markedly enhanced 24hUTP, whereas lower doses (5–100 mg/kg/day, *n* = 19) were 78.9% effective (Figure 10D).

## 4 DISCUSSION

### 4.1 Outcome profile and originality

Our meta‐analysis and systematic review, encompassing 42 in vivo studies on 793 animals, aimed to determine the therapeutic effects of resveratrol on kidney injury and its mechanisms in managing or delaying DN in rodent models. Key novel findings include the following: (i) resveratrol positively impacts renal pathology, kidney function, and metabolic parameters; (ii) radar map assessments offer detailed dosing recommendations, with resveratrol showing stronger effects at lower (≤15 mg/kg/day) and higher (100–200 mg/kg/day) dosages and weaker effects at moderate dosages (15–100 mg/kg/day); (iii) resveratrol exhibits antioxidative, autophagy‐stimulating, antihyperglycemic, antihyperlipidemic, endoplasmic reticulum (ER) stress‐reducing, anti‐inflammatory, and angiogenesis‐modulating properties; and (iv) the study provides a comprehensive, evidence‐based framework for translating preclinical findings to clinical applications.

The pathological characteristics of DN include GBM thickening, extracellular matrix accumulation, tubular sclerosis, and renal fibrosis. In DN meta‐analyses, the lack of standardized methods is a major hurdle to consolidating findings. Without uniform assessment, research outcomes are less comparable and replicable, limiting the full understanding of DN pathophysiology. Using standardized evaluation tools is vital for improving future study quality and reliability. This would aid data integration across studies and better inform clinical decisions and patient care. Future research should use consistent methodologies in multicenter prospective studies to enhance our understanding of DN and patient outcomes. The role of professional bodies in establishing and promoting these standards is key to scientific rigor and field progress.

The effects of resveratrol on CCr have shown inconsistent results, with some studies^24^, ^34^, ^38^, ^51^, ^57^ reporting an increase and others^41^, ^50^ observing a decrease. This variability may be attributed to the different stages of DN in the animal models used across studies. Specifically, Hao et al.^41^ utilized an animal model of STZ rats, administered a single intraperitoneal injection of STZ (50 mg/kg BW), and collected data 6 weeks postinjection while the rats were in a state of high filtration, where the CCr in the DN group was elevated compared to that in the model group, and SV intervention led to a decrease in CCr. Similarly, Park et al.^50^ studied 20‐week‐old db/db mice and noted that CCr was greater in these mice than in the db/m controls and observed a decrease in the CCr following resveratrol intervention. However, in the four studies, CCr in STZ‐injected rats treated with resveratrol at escalating doses (50 mg/kg at 4 weeks to 60 mg/kg at 16 weeks) was lower than that in controls. After resveratrol treatment, there was an improvement in CCr, suggesting that the animal models were in the later stages of DN.

Our meta‐analysis showed that resveratrol significantly reduces proteinuria, which is a key indicator of DN progression. However, the interpretation of these results must consider additional findings from several studies that included urine volume data. These studies revealed a decrease in urine volume in the resveratrol intervention group compared to the disease group, suggesting that it may have mitigated polyuria—a symptom of high filtration rates in DN—beyond its direct effect on proteinuria. Therefore, the observed reduction in 24hUTP excretion cannot be solely attributed to resveratrol without accounting for the potential decrease in urine volume, as this could also contribute to the lower measured proteinuria. To accurately isolate the effect of resveratrol on proteinuria and exclude the influence of changes in urine volume, it is essential to evaluate spot urine protein concentration measurements alongside volume assessments. This approach will provide a more nuanced understanding of the efficacy of resveratrol in managing proteinuria in DN patients.

Our meta‐analysis demonstrated that resveratrol can effectively control BG levels. However, among the studies included in the analysis, six^20^, ^22^, ^31^, ^43^, ^48^, ^52^ reported HbA1c levels. Notably, two studies^43^, ^48^ conducted within the intervention groups did not observe a reduction in HbA1c levels. One possible explanation for this discrepancy could be the relatively short duration of the resveratrol intervention in these studies, which ranged from 1 to 20 weeks, with the majority being less than or equal to 10 weeks. Since HbA1c reflects the average BG levels over the past 2–3 months, a shorter intervention period may not be sufficient to induce a significant change in HbA1c levels.

Additionally, the difference in the animal models used between these two studies and the others may also be a contributing factor. The two studies that did not report a decrease in HbA1c^43^, ^48^ utilized db/db mouse models, whereas the other four studies employed STZ‐rat models. The db/db mouse model is genetic and may have a different response to resveratrol than the STZ‐induced diabetic model, which is characterized by autoimmune destruction of pancreatic beta cells. Therefore, longer term studies with resveratrol intervention are needed to accurately assess its impact on HbA1c levels. Such research would provide a more comprehensive understanding of the effects of resveratrol on glycemic control and its potential therapeutic applications in diabetes management.

In future research, to assess the role of resveratrol more accurately, it may be necessary to adopt stricter experimental designs, including but not limited to randomized controlled trials with larger sample sizes, more precise measurement methods, and more comprehensive control of variables. Additionally, an in‐depth analysis of the existing study results may help reveal the underlying causes of the inconsistencies, thereby providing guidance for future research directions.

### 4.2 Possible cellular and molecular mechanisms of resveratrol against DN

#### 4.2.1 Promotion of antioxidant status

The protective effects of resveratrol against DN are multifaceted, primarily through the modulation of oxidative stress and advanced glycation end products. It alleviates early DN symptoms such as reduced creatinine and urea clearance, proteinuria, and oxidative stress by enhancing antioxidant enzyme activity and reducing lipid peroxidation, which is crucial for preventing renal dysfunction in DN patients. Additionally, resveratrol normalizes oxidative stress and inflammatory markers, suggesting that its renoprotective effect includes attenuating these stress markers and restoring the antioxidative Nrf2–Keap1 pathway in renal tissues.^29^, ^51^ This is evidenced by improved antioxidant defenses and normalized renal mRNA expression of various factors, indicating a broad impact on molecular pathways involved in DN progression.

Additionally, the influence of resveratrol on the SIRT1/FOXO3a pathway is crucial for its therapeutic potential in DN.^26^, ^27^ Increasing SIRT1 deacetylase activity and reducing acetylated FOXO3a expression^38^ mitigate oxidative stress induced by hyperglycemia, particularly in renal tubules. This pathway also plays a role in improving mitochondrial oxidative stress, as evidenced by decreased MDA content and MnSOD activity in the renal cortex of diabetic mice.^54^ The ability of this compound to restore the expression of SIRT1 and PGC‐1α further highlights its potential for reducing oxidative stress through multiple pathways.^39^

#### 4.2.2 Nephroprotective effect of resveratrol via autophagy stimulation

Resveratrol has demonstrated significant potential in protecting against DN through the stimulation of autophagy, a cellular process essential for the degradation and recycling of damaged cellular components. This protective effect is achieved through the modulation of key molecular players, such as microRNA‐383‐5p^56^ and the mTOR/ULK1 pathway,^55^ which are crucial for regulating autophagy. By enhancing autophagy, resveratrol not only prevents podocyte apoptosis but also reduces lipid deposition in nephrons, likely by decreasing lipogenic proteins and increasing lipidolysis‐related proteins.^61^

#### 4.2.3 Nephroprotective effect of resveratrol via reducing lipotoxicity

Lipotoxicity, marked by lipid accumulation in nonadipose tissues, significantly contributes to the development of DN. Resveratrol demonstrates nephroprotection by mitigating lipotoxicity, as shown by its reduction in kidney lipid levels in diabetic models. This effect correlates with increased AMPK phosphorylation^50^ and activation of the SIRT1–PGC‐1α pathway,^35^ both of which are crucial for cellular energy balance and are linked to preventing lipotoxic apoptosis and oxidative stress. Additionally, resveratrol upregulates AdipoR1 and AdipoR2,^45^ proteins critical for lipid metabolism, suggesting that it may enhance kidney function by modulating lipid profiles. Collectively, these findings underscore the therapeutic potential of resveratrol in addressing the metabolic imbalances underlying DN.

#### 4.2.4 Attenuation of endoplasmic reticulum stress and inflammation

ER stress and inflammation are key in DN pathogenesis. Resveratrol reduces the levels of ER stress markers, such as p‐PERK, GRP78, ATF4, and CHOP, in diabetic rats^31^ and is linked to improvements in DN indicators, suggesting that it enhances cellular stress adaptability. It also decreases the inflammatory factors PAI‐1, ICAM‐1, and NF‐κB, which are involved in renal inflammation and mesangial cell proliferation.^32^, ^48^ By inhibiting these pathways, resveratrol may prevent renal inflammation and fibrosis, which are characteristic of DN.^62^ These findings position resveratrol as a therapeutic agent targeting both ER stress and inflammation for DN management.

In conclusion, the nephroprotective effects of resveratrol against DN are multimechanistic and involve the modulation of oxidative stress, inflammation, autophagy, lipotoxicity, and ER stress. This finding underscores the potential of resveratrol as a therapeutic agent for DN, particularly due to its multitargeted approach. Further research is needed to clarify the underlying mechanisms and assess the clinical utility of resveratrol in DN management.

### 4.3 Safety and challenges of resveratrol administration

In our review of 42 studies, Xian et al.^30^ reported adverse outcomes, with two deaths in the T1DM (NOD mice) group and one in the resveratrol group after 8 weeks of intervention. This may be related to the choice of animal models, as different models can show varied responses to treatment and disease progression. In addition to NOD mice, STZ‐induced model rats and db mice are widely used for studying type 1 and type 2 diabetes, respectively, and are crucial for modeling human diabetic conditions. Genetically engineered models also significantly advance our understanding of DN at the molecular level. Choosing the right model is key to accurately reflecting human disease and improving research translation. Therefore, ongoing efforts to refine and diversify animal models to better mimic human DN complexities are essential for advancing our comprehension and treatment of this disease.

Resveratrol demonstrates dose‐dependent effectiveness in reducing BG levels and improving kidney injury biomarkers. At lower dosages of 2–5 mg/kg/day, it effectively lowers BG, suggesting that it is beneficial for glycemic control. However, a nuanced effect is observed in DN, where a dosage of 5–100 mg/kg/day shows decreased effectiveness. Notably, both low (≤15 mg/kg/day) and high (100–200 mg/kg/day) doses are equally effective at reducing BUN, suggesting a broad therapeutic window. Furthermore, the highest resveratrol doses provided the most significant improvements in 24hUTP, indicating a potential need for higher doses for greater renoprotection against albuminuria in DN.

Over the past 20 years, nearly 200 studies have explored the effects of resveratrol on various conditions, such as cancer, diabetes, and cardiovascular disease,^63^ with no consensus treatment regimens established, except for its general tolerance of up to 1 g/day.^64^ Resveratrol consistently reduces inflammation and improves metabolism. Our dose–response analysis supports the potential of resveratrol in treating DN, with both low and high doses showing promise, although individualized approaches are needed due to varied responses at intermediate doses. Resveratrol could be a valuable addition to DN treatment, but further optimal dosing research is needed.

### 4.4 Limitations

This review, while adhering to the PRISMA criteria, has limitations. This study, in its comprehensive analysis of the evidence for the impact of resveratrol on DN, encountered significant heterogeneity. This may be attributed to differences in treatment protocols, dosages, treatment durations, and research center settings. Such variability could affect the interpretation of our results and potentially undermine the generalizability of the findings. Therefore, we recommend a cautious approach in interpreting the results of this study and suggest that future research consider these potential sources of heterogeneity to improve the consistency of study design and the comparability of results. In addition, the assessment of certain effects was based on a limited number of studies, which limits the strength of the evidence for our findings. Our preliminary results need to be verified by a broader evidence base in future research. Selection bias may occur from focusing on four English‐language databases, potentially overlooking relevant research. High heterogeneity could result from varied study designs, including differences in animal models, methods, dosages, and schedules, affecting the interpretability of the results. Sensitivity analysis, Egger's test, and subgroup analyses supported the therapeutic efficacy of resveratrol for renal protection. The long‐term effects and safety profile of resveratrol are uncertain. Some studies omitted baseline characteristic details, and the absence of randomization in others raises methodological concerns. Future research needs a rigorous design and transparent reporting for reliable findings on the therapeutic potential of resveratrol. The early intervention timing in rodent models may not reflect clinical practice, and caution is needed when translating these findings.

## 5 CONCLUSION

In conclusion, our meta‐analysis and systematic review revealed that resveratrol is a promising therapeutic agent for DN due to its ability to improve renal function and reduce kidney injury in rodent models. This study indicated that resveratrol is effective across a spectrum of dosages, highlighting the importance of dosing optimization. Its nephroprotective mechanisms are multifaceted, targeting pathways related to oxidative stress, inflammation, autophagy, and metabolic regulation. Although the evidence is promising, further research is needed to refine dosing strategies, assess long‐term effects, and translate preclinical findings into clinical practice.

## AUTHOR CONTRIBUTIONS

Xiaojing Liu contributed to the drafting of the manuscript. Xiaojing Liu and Shimin Jiang formulated the search strategy and searched the databases. Xiaojing Liu and Jiao Zhang screened the articles and extracted the data. Xiangmeng Li and Xiansen Wei conducted the data analysis and quality assessment. Xia Gu and Wenge Li designed the research and revised the manuscript.

## FUNDING INFORMATION

This work was supported by grants from the National High Level Hospital Clinical Research Funding (2023‐NHLHCRF‐yS‐01) and the National Natural Science Foundation of China (82300815).

## CONFLICT OF INTEREST STATEMENT

The authors declare no conflicts of interest.^13^

## Supporting information


**Data S1.** Supporting information.

**Data S2.** PRISMA checklist.

## Data Availability

All data relevant to the study are included in the article or uploaded as Supporting Information.
