# Supplementary material for: Resveratrol delays the progression of diabetic nephropathy through multiple pathways: A dose–response meta‐analysis based on animal models
Source: J Diabetes. 2024 Sep 12;16(9):e13608. doi: 10.1111/1753-0407.13608 (PMC11391385; doi:10.1111/1753-0407.13608)
Supplement: Supplementary file 1 — Data S1. Supporting information. [file JDB-16-e13608-s001.docx]

**Supplementary Materials**

**Supplementary Figure legends**

Figure S1. Egger's and Begg's publication bias plots depicting publication bias and the trim-and-fill method.

Figure S2. Results of the sensitivity analysis. Meta-analysis estimates when each study was removed.

Figure S3. Forest plot (effect size and 95% CI) summarizing the effect of resveratrol on kidney functional parameters. A: The overall effect of resveratrol on urine volume. B: The overall effect of resveratrol on Alb.

Figure S4 Forest plot (effect size and 95% CI) summarizing the effect of resveratrol on metabolic parameters. A: The overall effect of resveratrol on the BW. B: The overall effect of resveratrol on KW. C: The overall effect of resveratrol on SBP.

Figure S5 Forest plot (effect size and 95% CI) summarizing the effect of resveratrol on biochemical parameters. A: the overall effect of resveratrol on TC. B: the overall effect of resveratrol on TG. C: the overall effect of resveratrol on LDL-C. D: the overall effect of resveratrol on HDL-C.

**Supplementary Table legends**

Appendix 1. Retrieval strategies

Appendix 2. Abbreviations

Table S1. Stratified analysis of pooled estimates according to BUN

Table S2. Stratified analysis of pooled estimates according to BG

Table S3. List of the molecular and cellular mechanisms driving the protective effect of resveratrol against DN identified in all 42 studies.


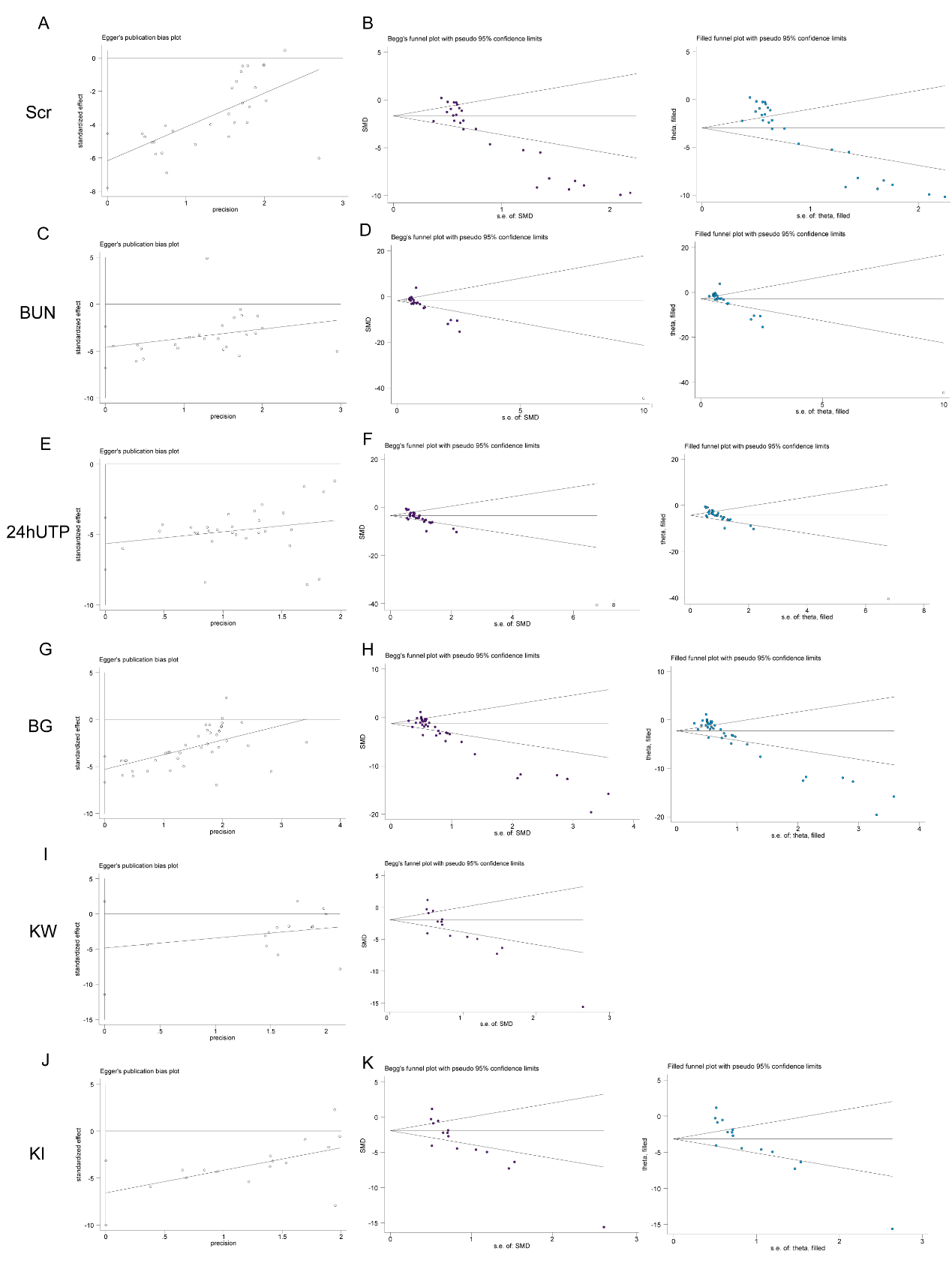


**Supplementary Figure 1** Egger's and Begg's publication bias plots depicting publication bias and the trim-and-fill method.


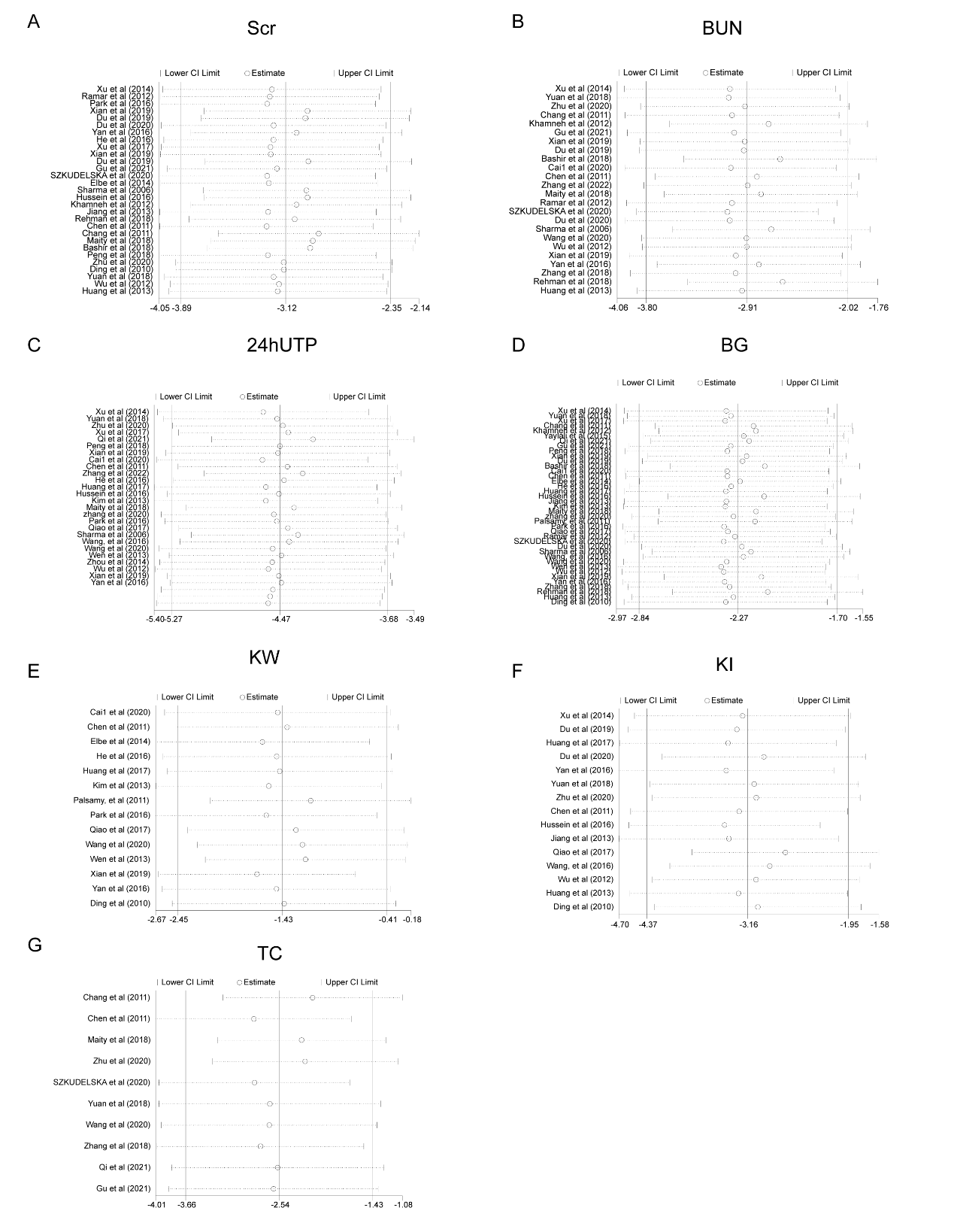


**Supplementary Figure 2.** Results of the sensitivity analysis. Meta-analysis estimates when each study was removed.


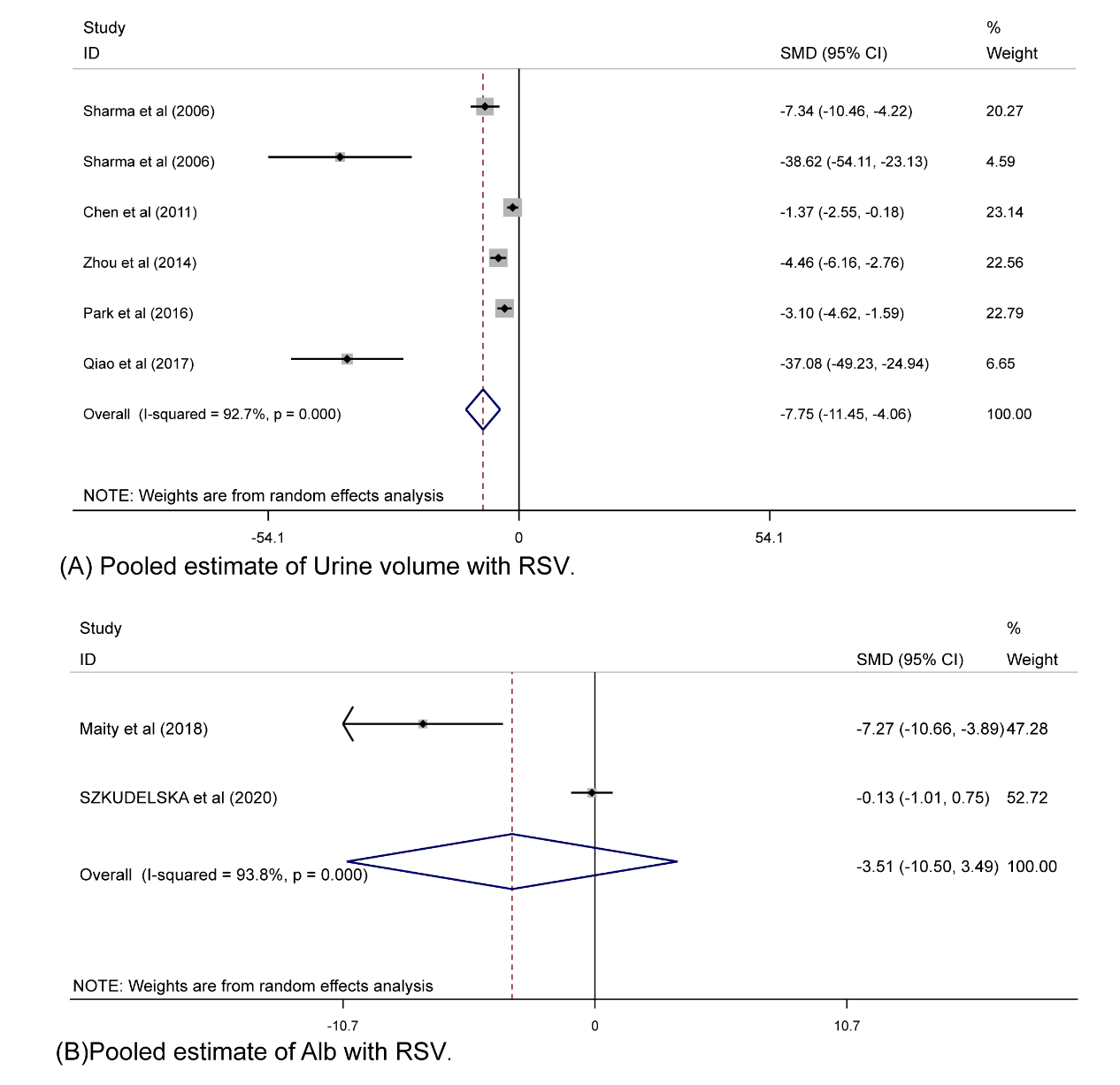


**Supplementary** **Figure 3** Forest plot (effect size and 95% CI) summarizing the effect of resveratrol on kidney functional parameters. A: Overall effect of resveratrol on the urine volume. B: Overall effect of resveratrol on Alb.


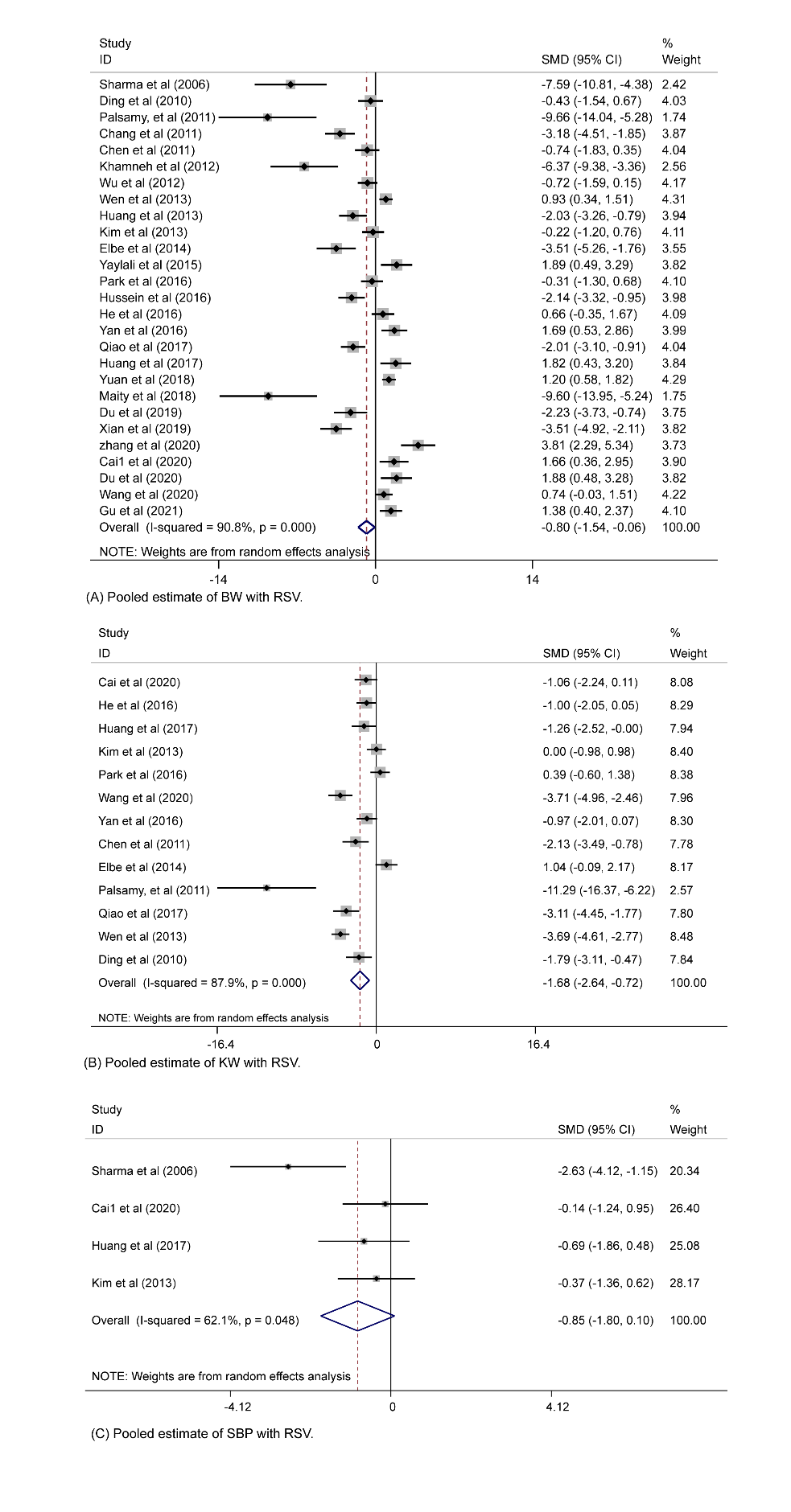


| **Supplementary Figure 4** Forest plot (effect size and 95% CI) summarizing the effect of resveratrol on metabolic parameters. A: the overall effect of resveratrol on BW. B: the overall effect of resveratrol on KW. C: the overall effect of resveratrol on SBP.  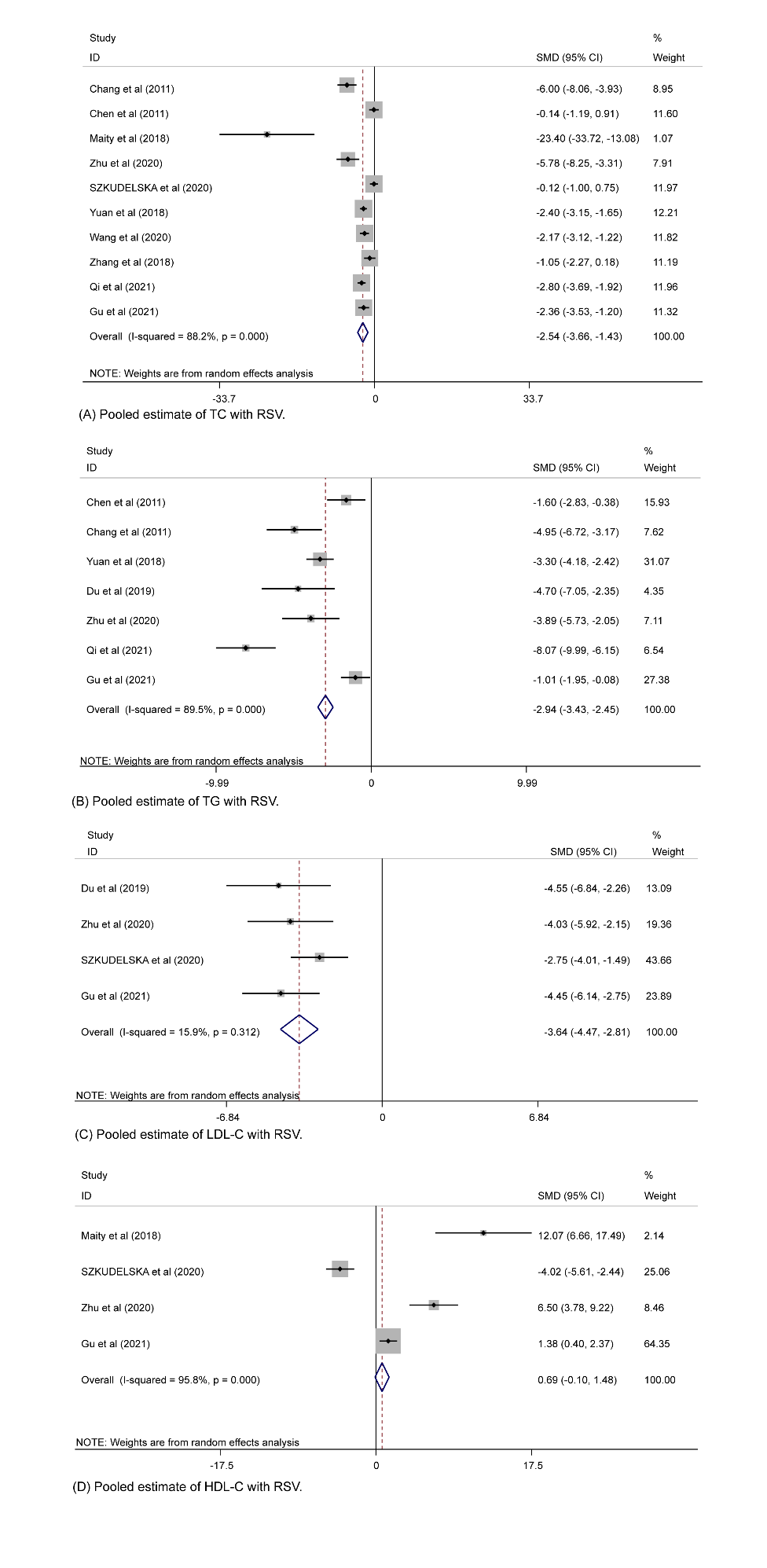  **Supplementary Figure 5** Forest plot (effect size and 95% CI) summarizing the effect of resveratrol on biochemical parameters. A: the overall effect of resveratrol on TC. B: the overall effect of resveratrol on TG. C: the overall effect of resveratrol on LDL-C. D: the overall effect of resveratrol on HDL-C.  ***Appendix 1. Retrieval strategies*** | | |
| --- | --- | --- |
| **Database** | **Number** | **Search terms** |
| PubMed | #1 | (Diabetic Nephropathy [mh]) OR (Nephropathies, Diabetic) OR (Nephropathy, Diabetic) OR (Diabetic Nephropathy) OR (Diabetic Kidney Disease) OR (Diabetic Kidney Diseases) OR (Kidney Disease, Diabetic) OR (Kidney Diseases, Diabetic) OR (Diabetic Glomerulosclerosis) OR (Glomerulosclerosis, Diabetic) OR (Intracapillary Glomerulosclerosis) OR (Nodular Glomerulosclerosis) OR (Glomerulosclerosis, Nodular) OR (Kimmelstiel-Wilson Syndrome) OR (Kimmelstiel Wilson Syndrome) OR (Syndrome, Kimmelstiel-Wilson) OR (Kimmelstiel-Wilson Disease) OR (Kimmelstiel Wilson Disease) |
|  | #2 | (resveratrol [mh]) OR (trans resveratrol) OR (trans-resveratrol) OR (cis-resveratrol) OR (cis resveratrol) OR (trans resveratrol 3 O sulfate) OR OR (3,5,4'-Trihydroxystilbene) OR (3,4',5-Trihydroxystilbene) OR (3,4',5-Stilbenetriol) OR (trans-resveratrol-3-O-sulfate) OR (SRT 501) OR (SRT501) OR (SRT-501) OR (resveratrol, (Z)-) OR (resveratrol-3-sulfate) OR (resveratrol 3 sulfate) |
|  | #3 | #1 AND #2 |
| **EMBASE** | #1 | diabetes nephropathy:ti,ab,kw OR diabetic glomerulopathy:ti,ab,kw OR diabetic glomerulosclerosis:ti,ab,kw OR diabetic intercapillary glomerulosclerosis:ti,ab,kw OR diabetic kidney disease:ti,ab,kw OR diabetic nephropathies:ti,ab,kw OR diabetic nephrosclerosis:ti,ab,kw OR glomerulonecrosis, intercapillary:ti,ab,kw OR glomerulosclerosis, diabetic:ti,ab,kw OR glomerulosclerosis, intercapillary:ti,ab,kw OR intercapillary glomerulosclerosis:ti,ab,kw OR kimmelstiehl wilson syndrome:ti,ab,kw OR kimmelstiel wilson disease:ti,ab,kw OR kimmelstiel wilson nephropathy:ti,ab,kw OR kimmelstiel wilson syndrome:ti,ab,kw OR nephropathy, diabetic |
|  | #2 | resveratrol/exp |
|  | #3 | (trans resveratrol):ti,ab,kw OR (trans-resveratrol):ti,ab,kw OR (cis-resveratrol):ti,ab,kw OR (cis resveratrol):ti,ab,kw OR (trans resveratrol 3 O sulfate):ti,ab,kw OR (SRT501):ti,ab,kw OR (SRT-501):ti,ab,kw OR (SRT 501):ti,ab,kw OR (resveratrol 3 sulfate):ti,ab,kw |
|  | #4 | Diabetic Nephropathies/exp |
|  | #5 | #1 OR #4 |
|  | #6 | #2 OR #3 |
|  | #7 | #5 AND #6 |
| **Web of Science** | #1 | (TS=((Diabetic Nephropathy) OR (Nephropathies, Diabetic) OR (Nephropathy, Diabetic) OR (Diabetic Nephropathy) OR (Diabetic Kidney Disease) OR (Diabetic Kidney Diseases) OR (Kidney Disease, Diabetic) OR (Kidney Diseases, Diabetic) OR (Diabetic Glomerulosclerosis) OR (Glomerulosclerosis, Diabetic) OR (Intracapillary Glomerulosclerosis) OR (Nodular Glomerulosclerosis) OR (Glomerulosclerosis, Nodular) OR (Kimmelstiel-Wilson Syndrome) OR (Kimmelstiel Wilson Syndrome) OR (Syndrome, Kimmelstiel-Wilson) OR (Kimmelstiel-Wilson Disease) OR (Kimmelstiel Wilson Disease)) AND TS=((resveratrol) OR ( 3,5,4'-trihydroxystilbene ) OR ( 3,4',5-trihydroxystilbene ) OR ( 3,4',5-stilbenetriol ) OR ( trans-resveratrol-3-O-sulfate ) OR ( trans resveratrol 3 O sulfate ) OR ( SRT 501 ) OR ( SRT501 ) OR ( SRT-501 ) OR ( 501-36-0 ) OR ( cis-resveratrol ) OR ( cis resveratrol ) OR ( trans-resveratrol ) OR ( trans resveratrol ) OR ( resveratrol-3-sulfate ) OR ( resveratrol 3 sulfate )) |
| **Cochrane Library** | #1 | MeSH descriptor: [Diabetic Nephropathies] this term only |
|  | #2 | (diabetic nephropathy or diabetic glomerulopathy or diabetic glomerulosclerosis or diabetic intracapillary glomerulosclerosis or diabetic kidney disease or diabetic nephropathies or diabetic nephrosclerosis or glomerulonecrosis, intracapillary or glomerulosclerosis, diabetic or glomerulosclerosis, intracapillary or intracapillary glomerulosclerosis or Kimmelstiehl Wilson syndrome or kimmelstiel wilson disease or kimmelstiel Wilson nephropathy or kimmelstiel wilson syndrome or nephropathy, diabetic):ti,ab,kw |
|  | #3 | #1 OR #2 |
|  | #4 | MeSH descriptor: [resveratrol] this term only |
|  | #5 | (trans-resveratrol-3-O-sulfate or trans resveratrol 3 O sulfate or SRT 501 or SRT501 or SRT-501 or cis-resveratrol or cis resveratrol or trans-resveratrol or trans resveratrol or resveratrol 3 sulfate) :ti,ab,kw |
|  | #6 | #4 OR #5 |
|  | #7 | #3 AND #6 |

| ***Appendix 2. Abbreviations*** | |
| --- | --- |
| ***Abbreviation*** | ***Full Name*** |
| 4E-BP1 | eukaryotic translation initiation factor 4e binding protein 1 |
| 95%CI | 95% confidence interval |
| ACEI | angiotensin-converting enzyme inhibitor |
| AdipoR1 | adiponectin receptor 1 |
| AdipoR2 | adiponectin receptor 2 |
| AGEs | advanced glycation end products |
| AKI | acute kidney injury |
| AMPK | activated protein kinase |
| ARB | angiotensin receptor blocker |
| ATF4 | activating transcription factor 4 |
| BG | blood glucose |
| BUN | blood urea nitrogen |
| BW | body weight |
| C | control |
| CAT | catalase |
| CCr | creatinine clearance rate |
| CHOP | c/ebp homologous protein chop |
| CKD | chronic kidney disease |
| CPT-1 | carnitine palmitoyltransferase 1 |
| DN | diabetic nephropathy |
| DPP-4 | dipeptidyl peptidase 4 |
| EMT | epithelial–mesenchymal transition |
| ER | endoplasmic reticulum |
| ESRD | end-stage renal disease |
| Flk-1 | fetal liver kinase-1 (a receptor for vegf) |
| FOXOs | forkhead box o transcription factors |
| GBM | glomerular basement membrane |
| GLP-1 RAs | glucagon-like peptide 1 receptor agonists |
| GPx | glutathione peroxidase |
| GRP78 | 78 kda glucose-regulated protein |
| GSH-Px | glutathione peroxidase |
| GSI | glomerular sclerosis index |
| HbA1c | glycated haemoglobin |
| HDL-C | high-density lipoprotein cholesterol |
| I | intervention |
| i.g. | oral gavage |
| i.p. | intraperitoneal injection |
| I^2^ | i-square |
| ICAM-1 | intercellular adhesion molecule 1 |
| IGF-1R | insulin-like growth factor 1 receptor |
| IL-1β | interleukin 1 beta |
| IL-6 | interleukin 6 |
| JAML/Sirt1 | juxtamembrane and multiple lysine-rich regions/sirt1 |
| KW | kidney weight |
| LDL-C | low-density lipoprotein cholesterol |
| MDA | malondialdehyde |
| MnSOD | manganese superoxide dismutase |
| MRA | mineralocorticoid receptor antagonist |
| mTOR/ULK | mechanistic target of rapamycin/unc-51 like autophagy activating kinase |
| mTORC1 | mammalian target of rapamycin complex 1 |
| N | number |
| NFkB | nuclear factor kappa-light-chain-enhancer of activated b cells |
| NM | not mentioned |
| NOD | nonobese diabetic |
| Nrf2 | nuclear factor erythroid 2-related factor 2 |
| O | outcome |
| P | population |
| p.o. | per os/oral medication |
| PAI-1 | plasminogen activator inhibitor 1 |
| PCOS | polycystic ovary syndrome |
| PERK | protein kinase r-like endoplasmic reticulum kinase |
| PGC‐1α | peroxisome proliferator-activated receptor-gamma coactivator-1alpha |
| PPARα | peroxisome proliferator-activated receptor alpha |
| p-PERK | phosphorylated protein kinase r-like endoplasmic reticulum kinase |
| PRISMA | preferred reporting items for systematic reviews and meta-analyses |
| PROSPERO | international prospective register of systematic reviews |
| qd | every day |
| RAGE | receptor for advanced glycation end-products |
| RAS | renin-angiotensin system |
| ROCK | rho-associated coiled-coil-coil coiled-coil |
| ROS | reactive oxygen species |
| SIRT1 | silent information regulator 1 |
| Scr | serum creatinine |
| SD | standard deviation |
| SGLT2 | sodium glucose cotransporter 2 |
| SOD | superoxide dismutase |
| SREBP-1c | sterol regulatory element-binding protein 1c |
| STZ | streptozotocin |
| T1DM | type 1 diabetes mellitus |
| T2DM | type 2 diabetes mellitus |
| TC | total cholesterol |
| TG | triglycerides |
| TGF-β1 | transforming growth factor-β1 |
| Tie-2 | Tek family tyrosine kinase |
| TNF-a | tumour necrosis factor alpha |
| UACR | urinary albumin to creatinine ratio |
| UAER | urinary albumin excretion rate |
| ULK1 | unc-51 like autophagy activating kinase 1 |
| VEGF | vascular endothelial growth factor |

| \| Table S1. Stratified analysis of pooled estimates according to BUN \| \| \| \| \| \| \| \| --- \| --- \| --- \| --- \| --- \| --- \| --- \| \| Variables \| Experiments (n) \| Individuals (n) \| SMD \| 95%CI \| P value \| Heterogeneity \| \| **Single dose** \|  \|  \|  \|  \|  \|  \| \| 0-20 \| 9 \| 127 \| -3.397 \| (-5.076, -1.719) \| p = 0.000 < 0.01 \| χ^2^ = 67.49,  I^2^ = 88.1% \| \| 20-100 \| 11 \| 226 \| -3.194 \| (-4.773, -1.616) \| p = 0.000 < 0.01 \| χ^2^ = 134.96,  I^2^ = 92.6% \| \| 100-200 \| 4 \| 76 \| -2.142 \| (-2.753, -1.531) \| p = 0.000 < 0.01 \| χ^2^ = 3.28,  I^2^ = 8.7% \| \| **Total dose** \|  \|  \|  \|  \|  \|  \| \| 0-100 \| 7 \| 98 \| -4.376 \| (-6.759, -1.993) \| p = 0.001 < 0.01 \| χ^2^ = 63.21,  I^2^ = 90.5% \| \| 100-500 \| 13 \| 255 \| -2.927 \| (-4.249, -1.606) \| p = 0.000 < 0.01 \| χ^2^ = 140.00,  I^2^ = 91.4% \| \| >500 \| 4 \| 76 \| -2.142 \| (-2.753, -1.531) \| p = 0.000 < 0.01 \| χ^2^ = 3.28,  I^2^ = 8.7% \| \| **Species** \|  \|  \|  \|  \|  \|  \| \| Rats \| 12 \| 236 \| -5.002 \| (6.959, -3.044) \| p = 0.000 < 0.01 \| χ^2^ = 167.67,  I^2^ = 93.4% \| \| Mice \| 12 \| 193 \| -1.909 \| (-2.599, -1.219) \| p = 0.000 < 0.01 \| χ^2^ =39.47,  I^2^ = 72.1% \| \| **Modelling methods** \| \|  \|  \|  \|  \|  \| \| Genetic background \| 8 \| 141 \| -1.652 \| (-3.250, -0.055) \| p = 0.043 < 0.05 \| χ^2^ = 81.40,  I^2^ = 91.4% \| \| STZ/Alloxan \| 16 \| 288 \| -3.656 \| (-4.764, -2.547) \| p = 0.000 < 0.01 \| χ^2^ = 123.84,  I^2^ = 87.9% \| \| **Overall** \| 24 \| 429 \| -2.914 \| (-3.804, -2.024) \| p = 0.000 < 0.01 \| χ^2^ = 208.45,  I^2^ = 89.0% \|   Note: Abbreviations (see Appendix 2); p < 0.05 represents the significant difference.   \| Table S2. Stratified analysis of pooled estimates according to BG \| \| \| \| \| \| \| \| --- \| --- \| --- \| --- \| --- \| --- \| --- \| \| Variables \| Experiments (n) \| Individuals (n) \| SMD \| 95%CI \| P value \| Heterogeneity \| \| **Single Dose** \| \|  \|  \|  \|  \|  \| \| 0-20 \| 15 \| 228 \| -3.086 \| (-4.225, -1.948) \| p = 0.000 < 0.01 \| χ² = 116.33,  I² = 88.0% \| \| 20-100 \| 18 \| 365 \| -1.616 \| (-2.337, -0.894) \| p = 0.000 < 0.01 \| χ² = 128.62,  I² = 86.8% \| \| 100-200 \| 6 \| 132 \| -2.842 \| (-4.319, -1.364) \| p = 0.000 < 0.01 \| χ² = 42.60,  I² = 88.3% \| \| **Total dose** \| \|  \|  \|  \|  \|  \| \| 0-100 \| 13 \| 229 \| -3.089 \| (-4.237, -1.941) \| p = 0.001 < 0.01 \| χ² = 100.31,  I² = 88.0% \| \| 100-500 \| 19 \| 344 \| -1.665 \| (-2.435, -0.896) \| p = 0.000 < 0.01 \| χ² = 141.07,  I² = 87.2% \| \| >500 \| 7 \| 152 \| -2.657 \| (-3.882, -1.432) \| p = 0.000 < 0.01 \| χ² = 42.86,  I² = 86% \| \| **Period** \|  \|  \|  \|  \|  \|  \| \| ≤8 W \| 22 \| 446 \| -3.372 \| (-4.247, -2.497) \| p = 0.000 < 0.01 \| χ² = 124.70,  I² = 87.2% \| \| >8 W \| 17 \| 279 \| -1.019 \| (-1.633, -0.405) \| p = 0.001< 0.01 \| χ² = 108.94,  I² = 90.8% \| \| **Species** \|  \|  \|  \|  \|  \|  \| \| Rats \| 21 \| 408 \| -2.707 \| (-3.572, -1.842) \| p = 0.000 < 0.01 \| χ² = 158.66,  I² = 87.4% \| \| Mice \| 18 \| 317 \| -1.897 \| (-2.665, -1.128) \| p = 0.000 < 0.01 \| χ² = 143.24,  I² = 88.1% \| \| **Overall** \| 39 \| 725 \| -2.274 \| (-2.843, -1.704) \| p = 0.000 < 0.01 \| χ² = 307.61,  I² = 87.6% \|   Note: Abbreviations (see Appendix 2); p < 0.05 represents the significant difference.  Table S3. ***List of molecular mechanisms driving the protective effect of resveratrol against DN in all 42 studies.*** | | | |
| --- | --- | --- | --- | --- | --- | --- | --- | --- | --- | --- | --- | --- | --- | --- | --- | --- | --- | --- | --- | --- | --- | --- | --- | --- | --- | --- | --- | --- | --- | --- | --- | --- | --- | --- | --- | --- | --- | --- | --- | --- | --- | --- | --- | --- | --- | --- | --- | --- | --- | --- | --- | --- | --- | --- | --- | --- | --- | --- | --- | --- | --- | --- | --- | --- | --- | --- | --- | --- | --- | --- | --- | --- | --- | --- | --- | --- | --- | --- | --- | --- | --- | --- | --- | --- | --- | --- | --- | --- | --- | --- | --- | --- | --- | --- | --- | --- | --- | --- | --- | --- | --- | --- | --- | --- | --- | --- | --- | --- | --- | --- | --- | --- | --- | --- | --- | --- | --- | --- | --- | --- | --- | --- | --- | --- | --- | --- | --- | --- | --- | --- | --- | --- | --- | --- | --- | --- | --- | --- | --- | --- | --- | --- | --- | --- | --- | --- | --- | --- | --- | --- | --- | --- | --- | --- | --- | --- | --- | --- | --- | --- | --- | --- | --- | --- | --- | --- | --- | --- | --- | --- | --- | --- | --- | --- | --- | --- | --- | --- | --- | --- | --- | --- | --- | --- | --- | --- | --- | --- | --- | --- | --- | --- | --- | --- | --- | --- | --- | --- | --- | --- | --- | --- | --- | --- | --- | --- | --- | --- | --- | --- | --- | --- | --- | --- | --- | --- | --- | --- | --- | --- | --- | --- | --- | --- | --- | --- | --- | --- | --- | --- | --- | --- | --- | --- | --- | --- | --- | --- | --- | --- | --- |
| Reference | Description | Outcome | Significance |
| **Nephroprotective effect of resveratrol by modulating oxidative stress and AGEs** | | | |
| Sharma et al (2006) | In vivo: resveratrol [5 and 10 mg/ (kg·d)] on renal function and oxidative stress in diabetic rats | resveratrol alleviated early signs of diabetic nephropathy (pre-DN), such as diminished creatinine and urea clearance, proteinuria, and oxidative stress markers like increased lipid peroxidation and reduced antioxidant enzyme activities. | The study reinforces the importance of antioxidant capacity of resveratrol against renal dysfunction in DN |
| Palsamy and Subramanian et al (2011) | In vivo: Renoprotective effect of resveratrol [5 mg/ (kg · d)] during diabetes | resveratrol normalized the levels of oxidative stress, inflammatory markers, renal expression of Nrf2/Keap1, and its downstream regulatory proteins in diabetic rats | This study demonstrates resveratrol’s renoprotective effect by attenuating oxidative stress markers and normalizing antioxidative Nrf2–Keap1 signalling in renal tissues of diabetic rats |
| Hussein et al (2016) | In vivo: resveratrol [5 mg/(kg·d)] on development and progression of DN in rats | resveratrol improved the antioxidant defence mechanism and normalized renal mRNA expressions of TGF-B1, fibronectin, NF-κB/p65, Nrf2, Sirt1, and FOXO1 | resveratrol's anti-DN effect is mediated through improving glycemic control and attenuating oxidative damage in kidneys |
| Wang et al (2017) | In vivo: resveratrol treatment [30 mg/(kg·d)] on hyperglycemia-induced oxidative stress in renal tubules in diabetic rats | resveratrol ameliorated renal dysfunction and glomerulosclerosis; it also increased SIRT1 deacetylase activity, while decreasing acetylated-FOXO3a expression and oxidative stress induced by hyperglycemia | resveratrol modulates the SIRT1/FOXO3a pathway by increasing SIRT1 deacetylase activity and ameliorates renal tubular oxidative damage. |
|  | In vitro: Effect of resveratrol treatment (25 μmol/L) on hyperglycemia-induced oxidative stress in human kidney epithelial cells |  |  |
| Wu et al (2012) | In vivo: Protective effect of resveratrol against DN in rats | resveratrol increased the expression of SIRT1 and FOXO1 activity; this was correlated with increased SOD activity, and decreased malondialdehyde, collagen IV, and fibronectin protein concentrations | resveratrol-mediated modulation of SIRT1/FOXO1 pathway may be a useful therapeutic target for treatment of DN |
| Bashir et al (2019) | In vivo: Combined administration of resveratrol [20 mg/ (kg · d)] and insulin against DN in rats | resveratrol and insulin synergistically increased renal cortex antioxidant enzyme activities, inhibited lipid peroxidation, and upregulated Na+/K+-ATPase, independent of each other | This study suggests that combined therapy with insulin and resveratrol may be an excellent therapeutic option for DN |
| Chang et al (2011) | In vivo: resveratrol 0.1 mg/kg (DM-R0.1) or resveratrol 1 mg/kg (DM-R1) on development and progression of DN in rats | resveratrol treatment reduced superoxide and protein carbonyls in diabetic kidneys, restored AMPK expression and phosphorylation, and altered inflammatory cytokine levels by decreasing IL-1b but increasing TNF-a and IL-6. | The study indicates that resveratrol combats oxidative stress, modulates inflammation, and boosts AMPK activity, potentially aiding in early-stage diabetic nephropathy protection. |
| Khamneh et al (2012) | In vivo: long-term(4-mongth) resveratrol (5 mg/kg/day) administration against DN in rats | Resveratrol boosts antioxidant defenses in diabetic rats by increasing glutathione, total antioxidant capacity, and activities of key enzymes (superoxide dismutase, glutathione peroxidase, and catalase). | The study suggests that chronic resveratrol administration is safe and effective and may be considered as a therapeutic compound in diabetes. |
| Qi et al (2021) | In vivo: resveratrol (150 mg/kg) alleviates renal damage and spermatogenesis issues in type 2 diabetic mice. | resveratrol mitigates oxidative stress and kidney damage by lowering MDA levels and enhancing SOD and GSH-Px activities. | The study suggests that resveratrol protects kidney tissue, normalizes blood glucose. |
| Elbe et al (2014) | In vivo: comparison of melatonin, quercetin, and resveratrol (10 mg/kg/day) in alleviating streptozotocin-induced diabetic nephropathy. | resveratrol reduced MDA levels and tGSH levels and SOD, CAT activities. | The study indicates that melatonin,  quercetin, and resveratrol might be helpful in reducing diabetes-induced renal damage |
| Du et al (2020) | In vivo: resveratrol (50 mg/kg) suppresses renal tubular cell EMT. | resveratrol improves kidney health by reducing EMT and fibrosis through Sirt1-mediated YY1 deacetylation. | The study provides new insights into effective therapeutic targets for DN and other kidney diseases associated with renal ﬁbrosis. |
| Wang et al (2020) | In vivo: resveratrol (10 mg/kg/day) attenuated oxidative stress and apoptosis in db/db mice; podocytes exposed to high glucose. | resveratrol reduces renal damage by inhibiting oxidative stress-induced podocyte apoptosis through AMPK activation. | This study reveales a possible mechanism to protect podocytes against apoptosis in DN. |
|  | In vitro: resveratrol inhibited oxidative stress production and apoptosis in human |  |  |
| Jiang et al (2013) | In vitro: resveratrol (20 mg/kg) attenuates early diabetic nephropathy, | resveratrol inhibited the proliferation of mesangial cells caused by high glucose and downregulated GSTM and Nrf2 expressions in a dose-dependent manner. | This study reveales resveratrol may help prevent DN progression by inhibiting high glucose-induced rat mesangial cell proliferation and reducing GSTM expression. |
|  | In vivo: effects on renal mesangial cells cultured In high glucose conditions. |  |  |
| Yan et al (2016) | In vivo: resveratrol (40 mg/kg) mitigates diabetic nephropathy in db/db mice; | These protective effects of resveratrol on DN were associated with the upregulation of HRD1, induced by resveratrol, and the promotion of IGF-1R ubiquitination and degradation | This study reveals that resveratrol induces HRD1 to enhance the ubiquitination and degradation of IGF-1R. |
|  | In vitro: protective effects In HKC-8 cells |  |  |
| Zhang et al (2018) | In vivo: resveratrol treatment in podocytes subjected to high glucose conditions; | resveratrol ameliorates podocyte damage in diabetic mice via SIRT1/PGC‐1α mediated attenuation of mitochondrial oxidative stress. | This study indicates that resveratrol mitigates podocyte injury in diabetic mice through SIRT1/PGC-1α-driven reduction of mitochondrial oxidative stress. |
|  | In vivo: The renoprotective effects of resveratrol (30 mg/kg/day) In diabetic mice. |  |  |
| Rehman et al (2018) | In vivo: Impact of resveratrol and Vit-E cotreatment on glycemia-regulated changes. | resveratrol decreased GSH, SOD and CAT enzymes activities. | This study indicates that resveratrol might offer a compelling option for future T2DM treatments. |
| Huang et al (2013) | In vivo: resveratrol (150 mg/kg) vs. Sirt1 In renal Injury amelioration. | Sirt1 activator resveratrol inhibited the expressions of FN and TGF-β1 induced by AGEs. | This study suggests resveratrol benefits DN through Nrf2/ARE pathway activation, enhancing antioxidant defense. |
| Ramar et al (2012) | In vivo: Ferulic acid vs. resveratrol (20 mg/kg) for diabetes protection in alloxan-treated mice. mice. | resveratrol likely mitigates liver, kidney, and pancreas damage in alloxan-induced diabetes by inhibiting the pro-inflammatory NFkB pathway. | The study shows that resveratrol possesses antioxidant and anti-diabetic effects. |
| **Nephroprotective effect of resveratrol by stimulating autophagy** | | | |
| Huang et al (2017) | In vivo: resveratrol (10 mg/kg) on autophagy in db/db mice | resveratrol regulated autophagy in *db/db* mice through suppressing microRNA-383-5p (miR-383-5p) expression | Activation of autophagy via miR-383-5p contributes to resveratrol's renoprotective effect in *db/db* mice |
|  | In vitro: resveratrol on autophagy in human podocytes |  |  |
| Zhu et al (2020) | In vivo: resveratrol [[5 and 10 mg/ (kg · d)] on autophagy In STZ rats | Res induced mTOR/ULK1-mediated autophagy and apoptosis and significantly reduced STZ mediated lipid deposition in nephrons, likely by decreasing the levels of lipogenic related proteins (SREBP-1c, ACS) and increased lipidolysis related proteins (PPARα, CPT-1). | The study shows the potential of Res in prevention of diabetic nephropathy. |
| Yaylali et al (2015) | In vivo: The renoprotective effects of resveratrol (30 mg/kg/day) in diabetic mice. | resveratrol ameliorates renal pathological injury and elevates leptin expression. | The study shows resveratrol improves renal pathological damage and enhances leptin expression independently of the sirtuin2 pathway. |
| Xu et al (2017) | In vivo: resveratrol [100 mg/ (kg · d)] on autophagy in DN in *db/db* mice | resveratrol increased LC3-II/LC3-I and synaptopodin expression while decreasing cleaved caspase 3; increased expression of autophagy related genes was positively correlated with miRNA-18a-5p expression | resveratrol-mediated autophagy induction via upregulation of miR-18a-5p/ATM is a potential therapeutic option for DN |
|  | In vitro: Effect of resveratrol (10 μmol/L) on autophagy-related genes in mouse podocytes |  |  |
| **Nephroprotective effect of resveratrol by reducing lipotoxicity** | | | |
| Kim et al (2013) | In vivo: resveratrol treatment on renal lipotoxicity and kidney function in *db/db* mice | resveratrol lowered lipid concentrations, which were correlated with increased AMPK phosphorylation and activation of SIRT1–PGC-1α signalling and of the key downstream effectors, PPARα–ERR-1α–SREBP1 | The study suggests that resveratrol helps prevent lipotoxicity-induced apoptosis and oxidative stress in the kidneys via activation of AMPK/SIRT1–PGC-1α signalling |
|  | In vitro: resveratrol (1, 5, 50 ng/mL) on glucotoxicity in mesangial (NMS2) cells |  |  |
| Park et al (2016) | In vivo: Preventive effect of resveratrol against DN in *db/db* mice | resveratrol increased phosphorylation of AMPK and SIRT1, decreased downstream effectors FOXO1 and FOXO3a by increasing AdipoR1 and AdipoR2 in renal cortex; it also increased expression of PPARγ coactivator-1α and estrogen-related receptor-1α, and decreased sterol regulatory element-binding protein 1 | The study suggests that resveratrol prevents DN by ameliorating lipotoxicity, oxidative stress, apoptosis, and endothelial dysfunction by increasing AdipoR1 and AdipoR2 expressions in kidney |
|  | In vitro: resveratrol on lipotoxocity in human glomerular endothelial cells (HGECs) | resveratrol prevented high-glucose–induced oxidative stress and apoptosis in glomerular endothelial cells by ameliorating lipotoxicity, which was evidenced by increased expression of AdipoR1 and AdipoR2 | The study indicates that resveratrol guards against lipotoxicity by reducing oxidative stress, apoptosis, and endothelial dysfunction. |
| Yaylali et al (2015) | In vivo: Effect of resveratrol (10 mg/kg) on Leptin and Sirtuin 2 expression in the kidneys in STZ-induced Diabetic Rats | resveratrol induced an increased expression of leptin in the diabetic kidney tissue | The study implies that resveratrol could prevent kidney damage from prolonged hyperglycemia through its antioxidant and anti-diabetic actions. |
| Gu et al (2021) | In vivo: resveratrol (100 mg/kg) improves diabetic kidney damage. | resveratrol regulates the JAML/Sirt1 lipid synthesis pathway, reduces lipid deposition in the kidney, and ameliorates diabetic kidney damage. | The study indicates that resveratrol reduces renal lipid buildup, improving diabetic kidney injury. |
| SZKUDELSKA et al (2020) | In vivo: resveratrol (20 mg/kg) impacts cholesterol accumulation in GK rats. | resveratrol reduces excessive cholesterol accumulation | The study shows that resveratrol positively affects cholesterol levels in diabetic rat tissues. |
| **Nephroprotective effect of resveratrol by attenuating ER stress and inflammation** | | | |
| Yuan et al (2018) | In vivo: resveratrol [50 mg/(kg · d)] on DN in diabetic rats | resveratrol decreased ER stress–associated signalling molecules p-PERK, GRP78, ATF4, and CHOP in kidneys, and these were correlated with amelioration in indicators of DN | The study suggests that resveratrol is a highly safe and effective agent against DN through its modulatory action on ER response in kidney cells |
| Xu et al (2014) | In vivo: resveratrol [10 mg/(kg · d)] on DN in diabetic mice | resveratrol decreased the expression of PAI-1 and intercellular adhesion molecule 1 while decreasing p-Akt/Akt ratio and NF-κB in the kidneys of diabetic rats; it also significantly decreased the density of PCNA-positive cells in glomeruli | This study indicates that resveratrol helps prevent DN by inhibiting renal inflammation via Akt/NF-κB pathway |
|  | In vitro: resveratrol on hyperglycemia-induced mesangial cell proliferation and inflammation | resveratrol attenuated high-glucose–induced PAI-1 expression and mesangial cell proliferation while inhibiting Akt and NF-κB activation | Anti-inflammatory effect of resveratrol in mesangial cells is likely mediated via inhibition of Akt/NF-κB pathway |
| Peng et al (2018) | In vivo: Coadministration of ramipril and resveratrol (15 mg/kg/day) for early-stage diabetic nephropathy glomerulosclerosis in DN rats | resveratrol reduced DN progression inearly stage DN glomerulosclerosis byinhibiting the RhoA/ROCK signalling pathway rather than the TGF-β signalling pathway. | This study suggests that resveratrol is an efficient strategy for early intervention in diabetes or diabetic nephropathy (DN). |
| Du et al (2019) | In vivo: resveratrol at 40 mg/kg in high glucose-cultured mouse glomerular mesangial cells (GMCs) serves as a positive control for comparison with AB-38b. | resveratrol notably lowered NLRP3 inflammasome levels in diabetes by suppressing the ROS/TXNIP/NLRP3 pathway and simultaneously enhanced Nrf2 signalling. | This study indicates that resveratrol enhances renal function in diabetic mice. |
| Xian et al (2019) | In vivo: Combined therapy with umbilical cord mesenchymal stem cells and resveratrol (200 mg/kg) protects against renal podocyte injury in NOD mice. | resveratrol reduced the expression of the inflammatory factors RAGE, NF-кB (P65) and NOX4 and improved the renal pathological structure. | This study indicates that the combination of Res and hUCMSCs may be a novel therapeutic method for the treatment of DN. |
| Cai et al (2020) | In vivo: The effects of resveratrol (10 mg/kg/day) on diabetic nephropathy in mice. | resveratrol improves intestinal barrier function and ameliorates intestinal permeability and inflammation by mediating changes in the gut microbiome, which provides supporting evidence for the gut–kidney axis in DN | The study suggests that resveratrol's gut microbiome modulation is a key component of its action, supporting the gut-kidney axis in diabetic nephropathy (DN). |
| Chen et al (2011) | In vivo: resveratrol (0.75 mg/kg) mitigates early-stage diabetic nephropathy. | resveratrol inhibited phosphorylation of smad2, smad3 and ERK1/2 in diabetic rat kidneys. | The study suggests that podocyte injuries of diabetic kidneys are lessened by resveratrol. |
| Zhang et al (2022) | In vivo: resveratrol at 20 mg/kg in STZ rat model serves as a positive control for comparison with Yishen Capsule. | Resveratrol decreased the expression of NLRP3, Caspase-1 and IL-1β in renal tissues | The study suggests that resveratrol can reduce microalbuminuria and alleviated pathological changes in DN rats, |
| Maity et al (2018) | In vivo: The combination of resveratrol (10 mg/kg/day) and vitamin D3 modulates proinflammatory cytokines in diabetic nephropathy in rats. | resveratrol reduced TNF-α and IL-6 expression in kidney tissue section. | The study suggests that Combined therapy with resveratrol and vitamin D3 is more effective for diabetic nephropathy than either alone, offering protective benefits for diabetic kidneys. |
| zhang et al (2020) | In vivo: resveratrol (40 mg/kg) protects kidneys in diabetic db/db mice. | resveratrol lowered ER stress markers GRP78, CHOP, and caspase-12 in mouse kidneys, and prevented apoptosis in nrK-52e cells, inhibiting HG-induced ER stress signalling in vitro. | The study indicates that resveratrol shields renal tubular cells from high glucose (HG)-induced apoptosis in DN through ER stress inhibition. |
|  | In vitro: resveratrol's impact on high glucose-induced apoptosis in renal tubular cells. |  |  |
| Qiao et al (2017) | In vitro: resveratrol's effect on high glucose-injured mesangial cells. | resveratrol reduced p38 MAPK activation and TGF-β1 expression | This study shows that resveratrol preserves renal tissue from diabetes-related harm, potentially through inhibiting the p38 MAPK/TGF-β1 pathway. |
|  | In vivo: resveratrol (20 mg/kg) protects kidneys in diabetic db/db mice. |  |  |
| Zhou et al (2014) | In vivo: resveratrol (50 mg/kg) improves diabetes-induced renal damage. | resveratrol Ameliorates Diabetes-induced Renal Damage through Regulating the Expression of TGF-β1, Collagen IV and Th17/Treg-related Cytokines in Rats | This study shows that resveratrol may mitigate diabetes-related kidney damage by regulating Th17/Treg cytokines, and curbing TGF-β1 and collagen IV expression. |
| **Nephroprotective effect of resveratrol by activating AMPK signalling pathway** | | | |
| Ding et al (2010) | In vivo: resveratrol treatment [10 mg/(kg · d)] on renal hypertrophy in early-stage diabetes in rats | resveratrol activated AMPK in rat kidneys and inhibited eukaryotic translation initiation factor 4E-BP1, and phospho-ribosomal protein S6 (S6), which was correlated with reduced plasma creatinine, urinary albumin excretion, and improved renal function | This study suggests that resveratrol protects against DN by activating AMPK and reducing 4E-BP1 and S6 phosphorylation |
|  | In vitro: resveratrol treatment (5, 10, 20 μmol/L) on rat renal mesangial cell proliferation |  |  |
| He et al (2016) | In vivo: resveratrol treatment [40 mg/(kg · d)] on renal interstitial fibrosis in DN of *db/db* mice | resveratrol treatment in *db/db* mice attenuated renal fibrosis, which was accompanied by an evident increase in p-AMPK and decrease in NOX4 | The study suggests that resveratrol is a potential therapeutic agent against diabetic renal fibrosis via regulation of AMPK/NOX4/ROS signalling |
|  | In vitro: resveratrol (20 μmol/L) on high-glucose–induced proliferation of rat kidney fibroblasts |  |  |
| **Nephroprotective effect of resveratrol by modulating angiogenesis** | | | |
| Wen et al (2013) | In vivo: Antiangiogenic activity of resveratrol [20 mg/ (kg · d)] against DN in rats | resveratrol decreased expression of VEGF, Flk-1, and angiopoietin 2, and increased expression of Tie-2 in rat kidneys, which was accompanied by improved kidney function | This study reinforces an important role of resveratrol's antiangiogenic activity in its beneficial effect on DN |
|  | In vitro: Antiangiogenic activity of resveratrol in mouse podocytes and endothelial cells |  |  |
| Note: Abbreviations (see Appendix 2). | | | |
